# Distribution of Pt single atom coordination environments on anatase TiO_2_ supports controls reactivity

**DOI:** 10.1038/s41467-024-45367-z

**Published:** 2024-02-02

**Authors:** Wenjie Zang, Jaeha Lee, Peter Tieu, Xingxu Yan, George W. Graham, Ich C. Tran, Peikui Wang, Phillip Christopher, Xiaoqing Pan

**Affiliations:** 1 Department of Materials Science and Engineering, University of California, Irvine, CA 92697 USA; 2 Department of Chemical Engineering, University of California, Santa Barbara, CA 93106 USA; 3 Department of Chemistry, University of California, Irvine, CA 92697 USA; 4 Department of Materials Science and Engineering, University of Michigan, Ann Arbor, MI 48109 USA; 5 Irvine Materials Research Institute, University of California, Irvine, CA 92697 USA; 6 Department of Chemistry, University of Sherbrooke, Sherbrooke, QC J1K 2R1 Canada; 7 Department of Physics and Astronomy, University of California, Irvine, CA 92697 USA

**Keywords:** Heterogeneous catalysis, Catalyst synthesis, Porous materials

## Abstract

Single-atom catalysts (SACs) offer efficient metal utilization and distinct reactivity compared to supported metal nanoparticles. Structure-function relationships for SACs often assume that active sites have uniform coordination environments at particular binding sites on support surfaces. Here, we investigate the distribution of coordination environments of Pt SAs dispersed on shape-controlled anatase TiO_2_ supports specifically exposing (001) and (101) surfaces. Pt SAs on (101) are found on the surface, consistent with existing structural models, whereas those on (001) are beneath the surface after calcination. Pt SAs under (001) surfaces exhibit lower reactivity for CO oxidation than those on (101) surfaces due to their limited accessibility to gas phase species. Pt SAs deposited on commercial-TiO_2_ are found both at the surface and in the bulk, posing challenges to structure-function relationship development. This study highlights heterogeneity in SA coordination environments on oxide supports, emphasizing a previously overlooked consideration in the design of SACs.

## Abstract (web summary)

Elucidating structure-function relationships is crucial for developing efficient catalysts. Here, the authors elucidate Pt single atom coordination environments on anatase TiO_2_ and correlate active site structure with CO oxidation activity.

## Introduction

Single-atom catalysts (SACs) have received significant interest due to the potentials for optimal precious metal utilization efficiency and tunable reactivity provided by control of active site local coordination environment^1–9^. Realization of these benefits could be facilitated by establishing structure-function relationships that relate active site structure (local coordination environment) to catalytic reactivity. While this goal may seem more feasible for SACs compared to supported metal nanoparticles (NPs), based on the known active site nuclearity, the likely existence of distributions of binding sites for single atoms (SAs) on supports introduces challenges^10–12^. This is particularly true for SACs on metal oxide supports, as oxide powders expose multiple surface facets, and each surface facet presents a range of SA binding sites^13^.

Given that most metal oxide supports used for SACs (except the simplest zeolites) exhibit a range of potential SA binding sites with energetically accessible formation energies^14–16^, the development of structure-function relationships require characterization of distributions of SA coordination environments^17^. However, most characterization tools are limited in probing such distributions. For example, imaging SAs coordination environments using scanning transmission electron microscopy (STEM) is typically statistically insignificant. X-ray absorption spectroscopy (XAS) provides sample averaged information and thus deconvolution into contributions from various coordination environments is typically statistically not feasible^18,19^. Probe molecule spectroscopies, such as infrared (IR) and nuclear magnetic resonance (NMR), can provide insights into the adsorption site distributions and the associated adsorption site structures^3,20^. However, probe molecule analyses are blind to sites that either bind molecules weakly or not at all.

SAC consisting of Pt SAs on anatase TiO_2_ supports have received attention in the context of catalytic performance and the development of active site models^3,4,8,21–30^. Characterization of low loadings of Pt (<0.1 wt.%) on anatase TiO_2_ surface via CO probe molecule IR spectroscopy has shown a narrow (6–8 cm^−1^ full width at half-maximum (FWHM) in Kubelka-Munk unit) band centered at ~2112 cm^−1,^^3,4,21^. Based on a correlation of IR measurements to density functional theory (DFT) calculations, along with support from STEM imaging and XAS fitting, this band was assigned to CO adsorbed to Pt^2+^ species in a strained square planar O coordination environment on either a stepped or terraced TiO_2_ (101) surface^31–33^. While the (101) surface is the thermodynamically most stable anatase TiO_2_ surface, anatase TiO_2_ particles typically expose both (101) and (001) surfaces, albeit with a predominance of (101) surfaces^33,34^.

The consistency between the experimental characterization results and theoretical predictions regarding the CO binding energy on Pt SAs (84 kJ/mol from experiments vs. 103 kJ/mol from theory) and the vibration frequency of CO bound to Pt SAs (2112 cm^−1^ from experiments vs. 2121 cm^−1^ from theory) indicates that the site responsible for CO adsorption in Pt/TiO_2_ SACs is Pt SAs in the +2 oxidation adsorbed on the (101) surface of anatase TiO_2_^21^. However, the question remains whether CO probe molecules are assessing all Pt atoms. Various Pt coordination environments could result in CO probe molecule IR being blind to those species. For example, Pt SAs on the CeO_2_(100) surface exhibit weak CO binding energies (~7 kJ/mol), as Pt is coordinatively saturated by lattice oxygen of CeO_2_ (Pt binding energy, 678 kJ/mol)^35,36^. Further, experimental evidence has suggested that Pt SAs can migrate to the subsurface of MgO after oxidation at 700 ^°^C and thus be inaccessible to CO^37,38^. It has also been suggested in several other studies that metal SAs can be located in the subsurface of oxides^39,40^. Hence, it remains an open question whether previous analysis of the Pt on anatase TiO_2_ SAC system have accounted for all Pt SAs when developing structure-function relationships.

In this study, we address this question by depositing Pt SAs onto shape-controlled anatase TiO_2_ particles, including TiO_2_-nanosheets exposing predominantly (001) surfaces and TiO_2_-truncated bipyramids exposing mainly (101) surfaces, and comparing the geometric locations, electronic properties, and catalytic activities of these Pt SAs to Pt SAs dispersed on commercial TiO_2_ particles. CO probe molecule Fourier transform infrared (FTIR) spectroscopy, X-ray photoelectron spectroscopy (XPS), depth-sectioning STEM, and CO oxidation kinetics suggest that Pt SAs prefer to localize on the (101) surfaces, as proposed previously, but that Pt instead prefers to reside under the (001) surfaces. Comparison of the kinetics for CO oxidation suggests that a non-negligible fraction of Pt SA resides in the sub-surface of commercial TiO_2_ supports, likely below the (001) surfaces. These results support previously proposed models of Pt SAs localized on (101) surfaces. However, the results also suggest that a certain fraction of Pt dispersed on commercial TiO_2_ support resides in the bulk, probably under the (001) facets. This study demonstrates the utility of shape-controlled supports for probing the distributions of metal coordination environments^41^, and highlights challenges associated with developing quantitative structure-function relationships for SACs.

## Results and discussion

### Synthesis of Pt/TiO_2_ SACs

**Fig. 1:**
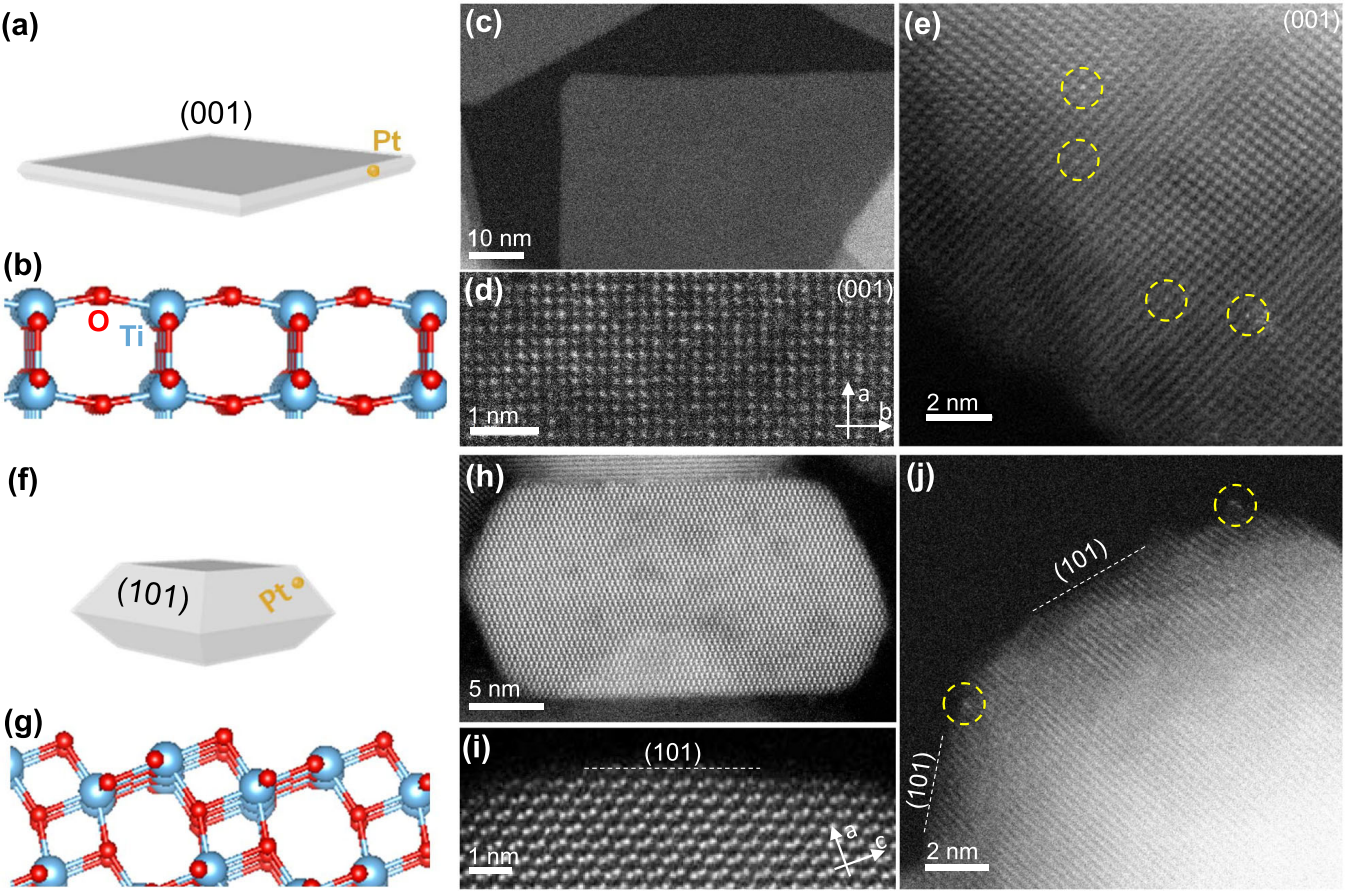
STEM images of Pt SAs dispersed on shape-controlled TiO_2_ supports. **a** Schematic illustration of Pt SAs dispersed on TiO_2_-nanosheet, in which dark gray surface represents the anatase (001) surface. **b** Corresponding atomic model for the (001) surface. Blue and red spheres represent Ti and O atoms. HAADF STEM images of TiO_2_-nanosheet (**c**, **d**) and Pt(0.05)/TiO_2_-nanosheet (**e**). **f** Schematic illustration of Pt SAs dispersed on TiO_2_-truncated bipyramid, in which light gray surface represents anatase (101) surface. **g** Corresponding atomic model for the (101) surface. HAADF-STEM images of TiO_2_-truncated bipyramid (**h**, **i**) and Pt(0.05)/TiO_2_-truncated bipyramid (**j**). The Pt SA is highlighted within the dotted yellow circle in (**e**) and (**j**).

To study the interaction between Pt SAs and different TiO_2_ surface facets, Pt was dispersed onto three TiO_2_ supports: commercial TiO_2_ nanoparticles (US Nano, surface area 226 m^2^/g), TiO_2_-nanosheets predominantly exposing (001) surfaces (Fig. 1a, b) (56 m^2^/g), and TiO_2_-truncated bipyramids mainly exposing (101) surfaces (Fig. 1f, g) (61 m^2^/g). Since the (001) surface is more reactive than the (101) surface due to the higher concentration of under-coordinated Ti centers, anatase TiO_2_ crystals synthesized without specific shape control efforts preferentially expose the less reactive (101) surface^42,43^. However, the inclusion of fluorine (F) during TiO_2_ synthesis can reverse the relative stability of (001) and (101) surfaces and suppress the growth of TiO_2_ crystals along [001] direction^42,43^. Thus, TiO_2_-nanosheets exposing primarily (001) facets were synthesized with high relative F concentration in solution, while TiO_2_-truncated bipyramids exposing primarily (101) facets was synthesized with low relative F concentration in solution (see Methods section for details on synthesis procedures). The potential influence of residual F on Pt coordination environment and reactivity is discussed below.

X-ray diffraction (XRD) confirms that the shape controlled TiO_2_ supports are in the anatase phase (Supplementary Fig. 1). High angle annular dark field (HAADF) STEM images confirm that TiO_2_-nanosheets mainly expose (001) surfaces (Fig. 1c, d), while TiO_2_-truncated bipyramids mainly expose (101) surfaces (Fig. 1h, i). Based on the shape and size of the TiO_2_ particles, it is estimated that around 90% of the TiO_2_-nanosheet surfaces are the (001) facet, while TiO_2_-truncated bipyramids expose the (101) surface with a 70% occupancy (Supplementary Fig. 2). Unlike shape-controlled TiO_2_ particles, it is difficult to discern the proportion of (001) and (101) surfaces on commercial TiO_2_ support, although STEM images suggest that both are present (Supplementary Fig. 3).

Electrostatic adsorption of Pt(NH_3_)_4_(NO_3_)_3_ from aqueous solutions was used to deposit Pt on TiO_2_ supports at weight loadings (wt.%) ranging from X = 0.025 to 1 wt.% Pt, denoted as Pt(X)/TiO_2_. After Pt deposition, Pt/TiO_2_-commercial was oxidized in air at 450 °C for 4 h, while Pt/TiO_2_-nanosheet and Pt/TiO_2_-truncated bipyramid were oxidized in air at 300 °C for 4 h. Pt/TiO_2_-nanosheet and -truncated bipyramid were calcined at 300 °C to avoid the deformation of shape-controlled TiO_2_ (Supplementary Fig. 4). Pt/TiO_2_-commercial was calcined at 450 °C to facilitate a direct comparison to results from our previous studies on this catalyst, although various analyses were also performed following 300 °C calcination^3,4,21^. A low loading of Pt (0.05 wt.%) was dispersed on the shape-controlled TiO_2_ supports to facilitate the deposition of primarily Pt SAs. Pt SAs were observed in HAADF-STEM images along the [001] projection of TiO_2_-nanosheet (Fig. 1e and Supplementary Fig. 5) and on the (101) surfaces of TiO_2_-truncated bipyramid (Fig. 1j and Supplementary Fig. 6). In our previous studies, we reported that Pt exists exclusively as SA (within the resolution of all characterization tools) on commercial TiO_2_ support at 0.025 wt.%, whereas Pt clusters are formed at the higher Pt loadings^3,4,21^. This is again confirmed in TEM images (Supplementary Fig. 7) and CO-IR spectra (Supplementary Fig. 8) (see Supplementary Discussion I for detail).

### Locating Pt SAs on shape-controlled TiO_2_ supports

As discussed above, previous analysis of Pt SAs on TiO_2_ has not addressed whether Pt is limited to adsorption on the TiO_2_ surface (1st layer). XPS is a surface-sensitive technique that probes the core level electrons of atoms located on the surface and sub-surface regions. The intensity of XPS signal decays exponentially as a function of depth. Thus, the XPS signal intensity of Pt located underneath the surface should be smaller than that of Pt located on the surface. Therefore, by quantifying the Pt XPS signal, it is possible to study the location of Pt SAs dispersed on the shape-controlled TiO_2_ supports. This approach is particularly advantageous here because the surface areas of TiO_2_-nanosheets (56 m^2^/g) and TiO_2_-truncated bipyramids (61 m^2^/g) samples are comparable. That is, the Pt density in the combined surface-subsurface volume should be similar at the same Pt loadings in Pt/TiO_2_ samples prepared with shape-controlled TiO_2_ supports.

**Fig. 2:**
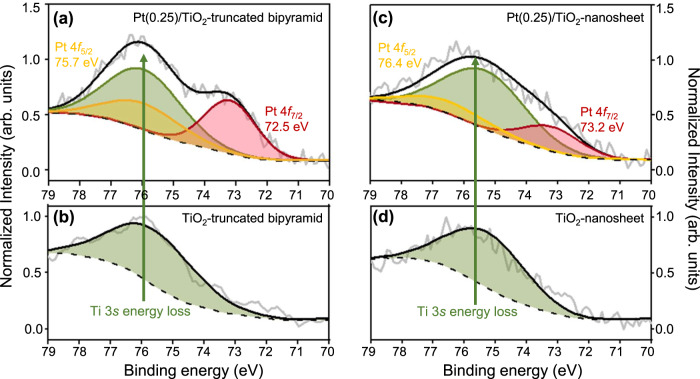
XPS spectra of Pt/TiO_2_. The XPS spectra of Pt(0.25)/TiO_2_-truncated bipyramid (**a**), TiO_2_-truncated bipyramid (**b**), Pt(0.25)/TiO_2_-nanosheet (**c**), and TiO_2_-nanosheet (**d**). All samples were ex-situ oxidized at 300 °C under flowing air (100 mL/min, 1 bar) before collecting the spectra. The spectra for Pt 4*f*_7/2_, Pt 4*f*_5/2_, and Ti 3 *s* are indicated by red, yellow, and green colors in the fitted data.

Figure 2 shows XPS spectra of TiO_2_-nanosheet and TiO_2_-truncated bipyramid samples with and without Pt, pre-oxidized at 300 °C in air for 4 h. It is challenging to identify the Pt 4 *f* signal from the XPS spectra in Pt/TiO_2_ samples with low Pt loading (0.05 wt.%), because the Pt 4 *f* signal overlaps with the signal from the Ti 3 *s* energy loss peak and the inelastic mean free path of Pt (1.5 nm) is shorter than that of Ti (2.8 nm). To enhance the XPS signal from Pt, the Pt loading was increased from 0.05 to 0.25 wt.% (Supplementary Fig. 9), where the Pt 4 *f* signal can clearly be distinguished from the Ti 3 *s* energy loss signal (Fig. 2). The relative ratio of Pt 4 *f* to Ti 3 *s* energy loss (Pt_4*f*_/Ti_3*s*_) is lower for Pt(0.25)/TiO_2_-nanosheet (0.50) than Pt(0.25)/TiO_2_-truncated bipyramid (1.04). Since the nominal Pt surface density for Pt(0.25)/TiO_2_-nanosheet (0.14 Pt atom/nm^2^) and Pt(0.25)/TiO_2_-truncated bipyramid (0.13 Pt atom/nm^2^) is similar, the difference in Pt_4*f*_/Ti_3*s*_ in these samples can be attributed to the differences in Pt location. Thus, XPS indicates that a higher fraction of Pt dispersed on TiO_2_-nanosheet is located below the surface, while on TiO_2_-truncated bipyramid, Pt is thought to be preferentially located on the surface. Also, the XPS signal from Pt increased when Pt(0.25)/TiO_2_-nanosheet was sputtered with an Ar_1000_ clusters at 10 keV, directed onto an area of 2 mm. This suggest that Pt atoms reside in TiO_2_ bulk when distributed on TiO_2_-nanosheet (Supplementary Fig. 10).

XPS also suggests that the average oxidation state of Pt in Pt(0.25)/TiO_2_-nanosheet, +2.8, is higher than that in Pt(0.25)/TiO_2_-truncated bipyramid, +1.9 (see Supplementary Figs. 11, 12 and Supplementary Discussion II). Note that only Pt SAs were observed on TEM images of Pt(0.25)/TiO_2_-nanosheet (Supplementary Fig. 13). While both Pt SAs and Pt-clusters were found on TEM images of Pt(0.25)/TiO_2_-truncated bipyramid, the abundance of Pt SAs is found to be higher than that of Pt-clusters (Supplementary Fig. 14). CO-IR spectra in Supplementary Fig. 15 indicates that the majority of Pt exists as SAs in Pt(0.25)/TiO_2_-nanosheet and Pt(0.25)/TiO_2_-truncated bipyramid, given a very weak IR intensity from CO bound to Pt clusters. The average oxidation state of Pt SAs in Pt(0.025)/TiO_2_-commercial after in-situ oxidation (+2.6) previously estimated by in-situ XANES is similar to that of Pt SAs in Pt/TiO_2_-nanosheets estimated here from XPS (+2.8)^4^. Through previously reported DFT calculations coupled with EXAFS fitting, it was predicted that Pt SAs in Pt(0.025)/TiO_2_-commercial replace Ti cations with an oxygen coordination number of 6 following in-situ oxidation^4^. The similar average oxidation state of Pt SAs in Pt(0.025)/TiO_2_-commercial and Pt(0.25)/TiO_2_-nanosheet indicate that Pt SAs in Pt/TiO_2_-nanosheet replace Ti cations and coordinate to six oxygen atoms in the TiO_2_ lattice. Thus, the XPS spectra intensity and estimated oxidation state of Pt suggest that Pt resides primarily in the bulk for Pt(0.25)/TiO_2_-nanosheet and that more Pt likely resides at the surface of Pt(0.25)/TiO_2_-truncated bipyramid.

**Fig. 3:**
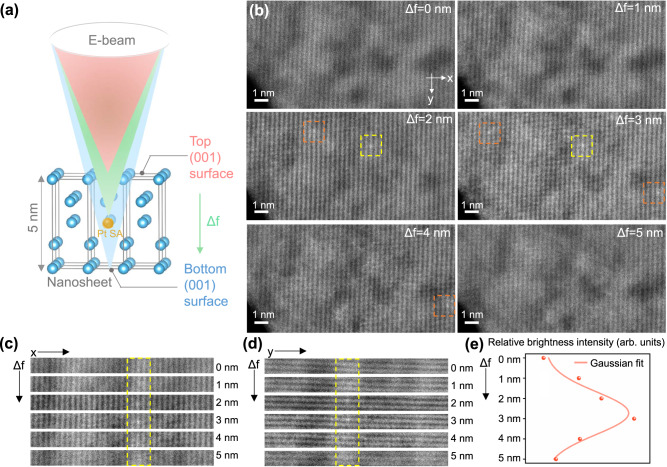
Three-dimensional analysis on the position of Pt SAs in Pt(0.25)/TiO_2_-nanosheet. **a** A schematic representation of how STEM images were collected at different focal planes along [001] direction. Blue and orange spheres represent Ti and Pt atoms, respectively. **b** Depth sectioning HAADF STEM images of Pt(0.25)/TiO_2_-nanosheet, revealing that abundant Pt SAs are present at the subsurface at the defocus value (Δf) of 2 nm. The Pt SA is highlighted within the dotted square. **c**, **d** A slice view of a Pt SA in x-Δf and y-Δf planes, showing that the Pt SA is situated in the middle of TiO_2_-nanosheet. **e** The brightness intensity profile from Pt SA relative to TiO_2_-nanosheet as the defocus value changes, along with the Gaussian fit.

To further assess whether Pt SAs dispersed on the (001) surfaces of TiO_2_ nanosheets reside in the TiO_2_ bulk, we performed a three-dimensional reconstruction of the individual Pt SAs deposited on a 5-nm-thick TiO_2_-nanosheet (Fig. 3). This was achieved by capturing a series of HAADF STEM images under a large illumination angle of 38 mrad with varying focus values through the TiO_2_-nanosheet sample with a known thickness of 5 nm (Fig. 3a). Figure 3b displays six images extracted through the focal series with a focal increment of 1 nm. As the electron beam approached the top surface, the crystalline structure of TiO_2_ began to appear, and this defocus value (Δf) was defined as 0 nm. No Pt SAs were observed when Δf = 0 nm, indicating the absence of Pt SAs on the surface. When the Δf was increased by 2 nm, two Pt SAs became visible, and with a further increase of Δf to 3 nm, three new Pt SAs were observed. As Δf was increased to 5 nm and approached the bottom TiO_2_ surface, the TiO_2_ crystalline structure was still apparent, but no Pt SA were observed. This observation supports the inference from XPS analysis that most Pt SAs in TiO_2_-nanosheet samples were situated approximately 2–3 nm below the (001) surface, instead of being on the top/bottom surfaces. It is worth mentioning that no beam damage was observed when comparing the morphology of the TiO_2_-nanosheet support in the same region before and after the focal series acquisition (Supplementary Fig. 16).

To provide a clear visualization of the three-dimensional position of Pt atoms in the TiO_2_-nanosheet, x-Δf and y-Δf slice views were extracted in Fig. 3c, d, based on six micrographs of one Pt SA (marked with a yellow dotted square) in the x-y plane shown in Fig. 3b. It is evident that the Pt SA is absent at both top and bottom surfaces, but its presence becomes increasingly visible in the middle of the Δf range (2–3 nm). This is confirmed by calculating brightness values of the Pt SA relative to the TiO_2_-nanosheet over the Δf range. According to Fig. 3e, when the electron beam was focused on Pt at the middle of the Δf range, the highest brightness was observed relative to the TiO_2_ support. These depth-sectioning STEM images confirm that Pt SAs reside ~2 nm below the (001) surface of nanosheets consistent with the XPS results.

Since TiO_2_-nanosheet was synthesized by fluorinating the (001) surface, it is worthwhile to discuss the potential interactions between F and Pt atoms in Pt/TiO_2_-nanosheet samples^22^. Supplementary Table 1 shows the F/Ti molar ratio of TiO_2_-nanosheet and Pt(0.25)/TiO_2_-nanosheet samples derived from XPS spectra. Residual F remained after multiple washings with water, ethanol, and acetone, and the amount is similar to previous reports^33,42,43^. Supplementary Fig. 17 shows the high-resolution F 1*s* XPS spectra of TiO_2_-nanosheet and Pt(0.25)/TiO_2_-nanosheet samples. Two F 1*s* peaks were observed at 684.3 eV and 682.0 eV in both samples, which are commonly associated with TiOF_x (x ≤ 2)_ or Ti-F species present on the surface, while we did not observe the XPS signal near 688.5 eV from F atoms doped into the bulk TiO_2_ lattice^33,42,43^. Therefore, XPS spectra suggest that F atoms are primarily present on the TiO_2_ surface, consistent with previous reports on the location of F atoms in TiO_2_-nanosheets^33,42^. Since XPS spectra (Fig. 2c, d) and TEM images collected at different focal points (Fig. 3) suggest that Pt atoms reside ~2 nm below the surface, it is hypothesized that F and Pt atoms do not interact directly in these samples. This implies that the bonding of Pt SAs within the TiO_2_ bulk is more favorable than bonding to F species, but further studies are needed to clarify this point.

XPS spectra and TEM images collected at different focal points show that the Pt SAs dispersed on the TiO_2_-nanosheet are located in the TiO_2_ bulk. This indicates that Pt SAs diffuse into the TiO_2_ bulk during the calcination treatment. Although the main focus of this study is to identify the distribution of Pt SAs binding sites on anatase TiO_2_ particles and the potential impact on catalytic activity, it is worth discussing how Pt SAs could diffuse into the bulk and why this would preferentially occur for TiO_2_-nanosheet.

As discussed  in relation to the XPS spectra in Fig. 2, Pt SAs present in Pt/TiO_2_-nanosheet may replace 6-coordinated Ti (Ti_6c_) positions in the TiO_2_ bulk after oxidation at 300 °C. Note that Pt SAs do not likely replace lattice O atoms, as the Pt atoms replacing lattice O atoms should be neutral or negatively charged^44^. Hence, Pt SAs should diffuse through Ti lattice in a similar way that Ti cations diffuse during phase changes. However, since the ionic radii of Pt ion (76.5 pm for Pt^4+^) is slightly larger than that of Ti^4+^ (74.5 pm), the replacement of Pt atoms with Ti atoms in the bulk would introduce stresses into the TiO_2_ structure that would likely cause an energetic penalty. Therefore, for Pt atoms to replace Ti atoms in the bulk, there should exist a pathway that can relieve the stress exerted on the TiO_2_ structure. The TiO_2_(001) surface is less stable than the (101) surface due to the higher concentration of undercoordinated Ti sites. To reduce the surface energy, the reactive (001) surface has been reported to reconstruct to a more stable (1 × 4)-(001) surface^45,46^. That is, unlike the thermodynamically stable (101) surface, the (001) surface is flexible in structure. This opens the possibility that the stress induced by the replacement of Pt atoms with Ti lattice atoms is relieved by the reconstruction of the (001) surface. This can potentially explain why only the Pt SAs dispersed on the (001) surface diffused to the TiO_2_ bulk.

These characterization studies show that the positions of Pt atoms differ depending on which TiO_2_ surface they are deposited on. When Pt SAs are deposited on the (101) surface, they preferentially localize at the surface. On the other hand, Pt SAs dispersed on the (001) surface prefer to migrate to the TiO_2_ bulk during thermal treatment. The distinct location of Pt atoms implies that the distribution of Pt SAs may not be uniform when dispersed on commercial TiO_2_ particles with non-uniform surface structures.

### Investigation of with which surface Pt SA dispersed in high surface area TiO_2_ mainly interacts

**Fig. 4:**
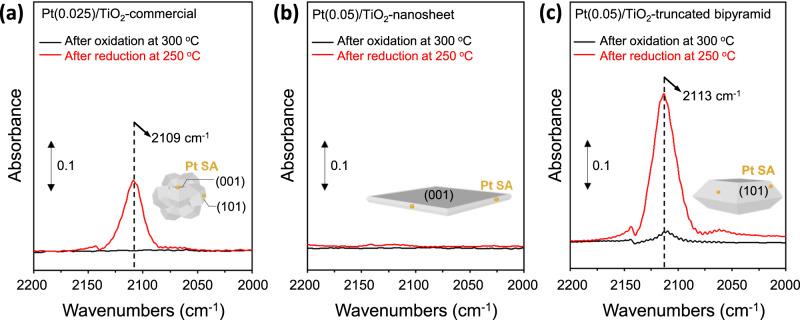
IR spectra of Pt/TiO_2_. FTIR spectra collected under 10% CO/Ar at 35 °C over (**a**) Pt(0.025)/TiO_2_-commercial, (**b**) Pt(0.05)/TiO_2_-nanosheet, and (**c**) Pt(0.05)/TiO_2_-truncated bipyramid after an oxidative pretreatment with pure O_2_ at 300 °C for 1 h or a reductive pretreatment with 10% H_2_/Ar at 250 °C for 1 h. The gas-phase CO signal was subtracted to identify the IR bands from CO adsorption on Pt.

We next consider the behavior of Pt SAs deposited on commercial TiO_2_ that contains both (101) and (001) surfaces (Supplementary Fig. 3). As discussed above, CO probe molecule IR spectroscopy provides sample-averaged and site-specific information, as the vibrational frequency of CO varies when adsorbed on metal sites with different coordination environments. Figure 4 shows CO probe molecule FTIR spectra of Pt(0.025)/TiO_2_-commercial, Pt(0.05)/TiO_2_-nanosheet and Pt(0.05)/TiO_2_-truncated bipyramid samples collected after in-situ oxidation with 100% O_2_ at 300 °C and reduction with 10% H_2_ at 250 °C. Since the crystallinity of TiO_2_ did not change after the reductive treatment (Supplementary Fig. 18), any changes in the IR band of CO bound to Pt SA after reductive treatment can be attributed to the changes in the coordination of Pt SAs.

After oxidative treatment, the IR intensity from CO bound to Pt SAs was not visible for Pt/TiO_2_-commercial and Pt/TiO_2_-nanosheet, and was very weak for Pt/TiO_2_-truncated bipyramid (Fig. 4). The lack of CO adsorption sites could be caused by weak binding of CO to Pt SA (the data was recorded at 35 °C where species binding with <~1 eV will be hard to resolve) or the inaccessibility of CO to Pt SA located in the TiO_2_ bulk^4,38^. After H_2_ reduction and exposure of the samples to CO, the IR band from CO bound to Pt SA was observed at 2109 cm^−1^ in Pt/TiO_2_-commercial (Fig. 4a) and 2113 cm^−1^ in Pt/TiO_2_-truncated bipyramid (Fig. 4c)^3^. The appearance of these IR bands suggests that the Pt SA coordination was modified due to H_2_ treatment, as compared to the oxidation treatment, which is likely due to Pt oxidation state reduction and modified Pt-O coordination through water evolution (Pt reduction). The similar stretching frequencies of CO bound to Pt SA (2109 vs 2113 cm^−1^) indicate similar Pt coordination environments in these two samples. We note that small oxidized Pt clusters reduce in H_2_ at these conditions, and thus the lack of CO stretches at <2100 cm^−1^ suggests that the exposed Pt species primarily exist as Pt SAs^3^.

Interestingly, the IR band from CO adsorbed on Pt was not detected in Pt(0.05)/TiO_2_-nanosheet (Fig. 4b). This absence is noteworthy, especially when considering the CO-IR spectra (Supplementary Fig. 15a) and TEM images (Supplementary Fig. 19) of Pt(0.25)/TiO_2_-nanosheet, which, despite having a 5-fold higher Pt loading, indicates that the majority of Pt exists as SAs. This suggests that Pt SAs on TiO_2_-nanosheet are primarily inaccessible for CO adsorption. Since XPS and TEM analysis suggest that Pt deposited on the (101) facets of TiO_2_-truncated bipyramid primarily resides on the surface and a majority of Pt SAs deposited on the (001) facets of TiO_2_-nanosheet resides in the bulk, it can be concluded from Fig. 4 that Pt SAs that can be probed by CO are located on the (101) surface, and those that cannot be detected by CO reside in the TiO_2_ bulk. Figure 4 also indicates that a large fraction of Pt SAs dispersed on TiO_2_-commercial are located on the (101) surface, as we see a large absorbance from CO bound to Pt SA (maximum absorbance from CO bound to Pt SA is 0.139 at 0.025 wt.%, Fig. 4a), which is comparable to that of Pt/TiO_2_-truncated bipyramid considering the Pt loading (maximum absorbance from CO bound to Pt SA is 0.305 at 0.05 wt.%, Fig. 4c).

To further evaluate the distribution of Pt on (or under) different TiO_2_ surfaces, we compared CO-IR spectra of Pt/TiO_2_-nanosheet and Pt/TiO_2_-truncated bipyramid with different Pt loadings. For Pt(0.25)/TiO_2_-nanosheet, an IR band of CO bound to Pt SA could be resolved, although the intensity is low (maximum absorbance is 0.1 at 0.25 wt.%) compared to that on Pt/TiO_2_-truncated bipyramid (maximum absorbance from CO bound to Pt SA is 0.3 at 0.05 wt.%) (Supplementary Fig. 20). This indicates that a small fraction of Pt SAs are located on the (001) surface of TiO_2_-nanosheet and reached a detectable level by IR at 0.25 wt.% of Pt. On Pt(1)/TiO_2_-nanosheet, the IR intensity of CO bound to Pt SA slightly decreased and that of CO bound to Pt NP increased (identified based on CO stretches <2100 cm^−1^)^3^. This suggests that deposited Pt formed clusters at higher loadings. On Pt/TiO_2_-truncated bipyramid, the IR intensity of CO bound to Pt SA decreased and that of CO bound to Pt NP increased when the Pt loading increased from 0.05 to 0.25 wt.% (Supplementary Fig. 20b). This suggests that Pt present near the TiO_2_ surface sintered more readily than when Pt resided in the TiO_2_ bulk, when comparing the results to the TiO_2_-nanosheet behavior. When the Pt loading was increased to 1 wt.% on TiO_2_-truncated bipyramid, the IR intensity of CO bound to Pt NP increased, indicating that Pt primarily formed clusters. Comparing the IR spectra of Pt/TiO_2_-nanosheet and Pt/TiO_2_-truncated bipyramid (Supplementary Fig. 20), the IR intensity of CO bound to Pt SA decreased on Pt/TiO_2_-truncated bipyramid when Pt loading increased from 0.05 to above 0.25 wt.%, but it increased on Pt/TiO_2_-nanosheet. These contrasting trends again suggest that a large fraction of Pt deposited on the (001) surface of TiO_2_-nansheet is located in the bulk and cannot be detected by CO molecules.

Finally, to evaluate the uniformity of Pt SA coordination environments dispersed on TiO_2_ surfaces, the FWHM of CO bound to Pt SA of Pt/TiO_2_-commercial and Pt/TiO_2_-truncated bipyramid are compared with the values reported in the literature (Supplementary Table 2)^3,4,25,47^. When studying SACs using CO-IR spectra, the presence and nature of SAs have been commonly discussed based on the CO stretch peak position. However, less attention has been paid to evaluating the FWHM of CO bound to SA, which conveys useful information about the uniformity of SA binding sites^48^. While a narrow CO-IR band indicates that SAs being probed by CO are uniformly distributed with similar coordination to the support, it is worth emphasizing that the FWHM is also affected by the unit used in the IR spectra, and thus, it is preferable to specify the unit when comparing the FWHMs (Supplementary Fig. 21)^20,49^. The FWHM of CO bound to Pt SA on Pt/TiO_2_-commercial (~20 cm^−1^ in Absorbance and 13 cm^−1^ in KM) observed here is slightly larger than what we previously reported (~11 cm^−1^ in Absorbance and 6–10 cm^−1^ in KM) (Supplementary Table 2). Nevertheless, the FWHM of CO bound to Pt SA on Pt(0.025)/TiO_2_-commercial is similar to that of Pt(0.05)/TiO_2_-truncated bipyramid, 25 cm^−1^ in Absorbance unit and 17 cm^−1^ in KM unit (Supplementary Table 2). This demonstrates that the Pt SAs being probed by CO are in a similar coordination environment in these two samples.

Therefore, the CO-IR spectra indicate that: (i) Pt SAs detectable by CO probe molecule are distributed on the (101) surface, and that (ii) some Pt SAs cannot be probed by CO since Pt deposited on the (001) surface diffuse to the TiO_2_ bulk. CO-IR spectroscopy is frequently used to identify the presence of Pt SAs and characterize their electronic properties. However, our results suggest that caution should be exercised when studying SACs using CO-IR spectroscopy, as there may exist Pt SAs that cannot be probed with CO.

### CO oxidation reactivity of Pt/TiO_2_ catalysts

So far, we have learned that the Pt SAs dispersed on the (001) surface of anatase TiO_2_ can diffuse to the bulk. The CO-IR spectra also demonstrate that a large fraction of Pt SAs dispersed on commercial TiO_2_ are located on the (101) surface. However, we cannot rule out the possibility that some of the Pt SAs are located below the (001) surface, since these Pt SAs cannot be detected by CO probe molecule. Considering the potential heterogeneity in the Pt SA coordination environments in Pt/TiO_2_-commercial catalysts, we next compare the catalytic activities of Pt SAs dispersed on different TiO_2_ surfaces to further assess the distribution of Pt SAs coordination environments.

**Fig. 5:**
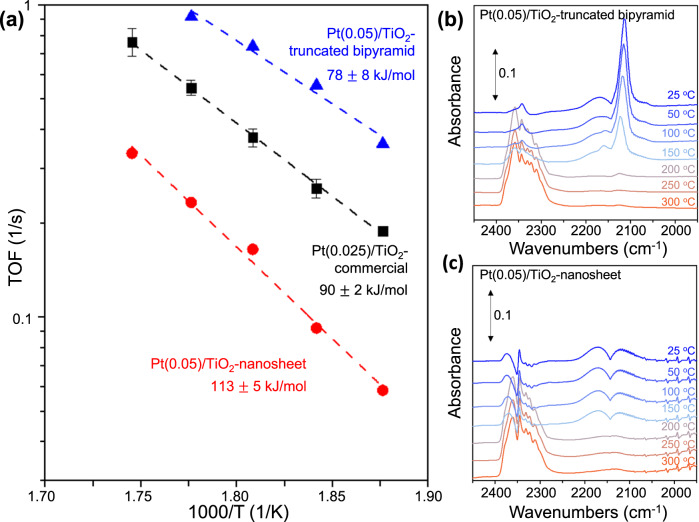
Comparison of CO oxidation activity of Pt/TiO_2_ catalysts. **a** The Arrhenius plots show the temperature dependence of the per-Pt-atom rate (1/s) for CO oxidation on each catalyst within the temperature range from 250 to 300 °C. The apparent activation energy (${E}_{a}$) is indicated for each catalyst, and the error bars in TOF represent the standard deviation from four independent measurements. *Operando* IR spectra of (**b**) Pt(0.05)/TiO_2_-truncated bipyramid and (**c**) Pt(0.05)/TiO_2_-nanosheet catalysts collected during CO oxidation at conditions identical to (**a**). A flow rate of 50 sccm comprising 1% CO, 10% O_2_, and balance Ar was used for the activity measurement and spectra collection.

To compare the catalytic activity of Pt SA dispersed on different TiO_2_ surfaces, CO oxidation was performed in a kinetically controlled region (Supplementary Fig. 22) for Pt(0.025)/TiO_2_-commercial, Pt(0.05)/TiO_2_-nanosheet and Pt(0.05)/TiO_2_-truncated bipyramid catalysts, in which Pt NP could not be detected in characterization studies^4^. Here, it is important to mention that catalytic activity measurements were carried out with a gradual decrease in reaction temperature from 300 ^o^C to 250 °C to ensure a consistent catalyst state throughout the reaction. Stable reactivity was observed at each temperature throughout the course of the experiment. Pt/TiO_2_-truncated bipyramid showed the highest activity (turnover frequency (TOF) of CO converted per Pt atom at 270 °C, 0.55 1/s), followed by Pt/TiO_2_-commercial (TOF at 270 °C, 0.26 1/s) and then Pt/TiO_2_-nanosheet (TOF at 270 °C, 0.09 1/s) (Fig. 5a). Interestingly, the apparent activation energy (${E}_{a}$) exhibited by Pt/TiO_2_-commercial catalyst for CO oxidation, 90 ± 2 kJ/mol, was close to that of Pt/TiO_2_-truncated bipyramid, 78 ± 8 kJ/mol, and lower than that of Pt/TiO_2_-nanosheet, 113 ± 5 kJ/mol. The ${E}_{a}$ of Pt/TiO_2_-nanosheet was similar to that of Pt-free TiO_2_-nanosheet (138 ± 4 kJ/mol, Supplementary Fig. 23). Note that the ${E}_{a}$ of Pt/TiO_2_-nanosheet for CO oxidation reaction is much higher than those of other Pt/TiO_2_ SACs where Pt SAs are believed to be located near TiO_2_ surface (Supplementary Table 3).

These results suggest that Pt/TiO_2_-truncated bipyramid and Pt/TiO_2_-commercial share similar active sites, likely Pt SAs on the (101) surface, while Pt SAs distributed on the (001) surface exhibit lower activity due to inaccessibility to the reactants. Previous studies suggest that metal atoms at the subsurface of an oxide can promote the reactivity of oxide surface^39,40^. However, it is likely that the Pt SAs dispersed in TiO_2_-nanosheet only weakly influence the catalytic reaction, likely because they are located too deep in the bulk (~2 nm from the surface). Also, the higher catalytic activity of Pt/TiO_2_-truncated bipyramid over Pt/TiO_2_-commercial further suggests that a larger fraction of Pt is located under the (001) surfaces in the Pt/TiO_2_-commercial sample.

In-situ CO-IR spectra were collected during CO oxidation to examine the state of Pt SA dispersed on different TiO_2_ surfaces (Fig. 5b, c). In Fig. 5a, CO oxidation was performed in the temperature range of 250 to 300 °C, but only the formation of gaseous CO_2_ was observed in the IR spectra above 200 °C for both Pt(0.05)/TiO_2_-truncated bipyramid and Pt(0.05)/TiO_2_-nanosheet catalysts. Interestingly, the IR band corresponding to CO bound to Pt SA was observed on Pt(0.05)/TiO_2_-truncated bipyramid (Fig. 5b) but not on Pt(0.05)/TiO_2_-nanosheet (Fig. 5c) when the temperature was below 150 °C. The appearance of the CO-IR band from Pt SA in Pt(0.05)/TiO_2_-truncated bipyramid suggests that the six-fold coordinated Pt SA on the surface, which binds CO weakly (Fig. 4c), is partially uncoordinated, likely forming the 4-fold coordinated Pt^2+^ species during the reaction^4^. This is consistent with our previous report that Pt SAs in oxidized and slightly reduced states on TiO_2_-commercial surface approach similar oxidation state under CO oxidation conditions^4^. On the other hand, even after CO oxidation at 300 °C for 2 h, Pt SAs dispersed on TiO_2_-nanosheet did not exhibit significant CO coverage (Fig. 5c). This suggests that most of the Pt SAs in the Pt/TiO_2_-nanosheet sample are located in the TiO_2_ bulk under CO oxidation conditions, which explains the comparable ${E}_{a}$ observed for Pt/TiO_2_-nanosheet and pure TiO_2_-nanosheet (Supplementary Fig. 23). Note also that we could not observe IR bands from CO bound to Pt clusters after CO oxidation reaction at 300 °C (Fig. 5). This indicates that Pt SAs did not sinter into clusters after the reaction at 300 °C within the resolution of our measurement.

Lastly, while F ions could not be completely removed by several washing or thermal treatments in Pt/TiO_2_-truncated bipyramid and Pt/TiO_2_-nanosheet catalysts (Supplementary Table 1), the CO-IR spectra and CO oxidation kinetics suggest that F ions in shape-controlled TiO_2_ particles do not play a direct role in determining the Pt SA coordination environment or the CO oxidation activity. For example, Pt/TiO_2_-truncated bipyramid and Pt/TiO_2_-commercial SACs exhibited similar CO oxidation kinetics (Fig. 5) and adsorbed CO stretching frequencies (Fig. 4), despite the presence of F ions in Pt/TiO_2_-truncated bipyramid SAC. If Pt atoms are indeed interacting with F ions, we would expect significantly different ${E}_{a}$ for CO oxidation reaction and vibrational frequency for CO bound to Pt SAs in Pt/TiO_2_-truncated bipyramid and Pt/TiO_2_-commercial samples. It is also worth noting that the vibrational frequencies of CO bound to Pt SAs observed in our study align well with those reported in the literature where Pt SAs are dispersed on F-free TiO_2_ surface (Supplementary Table 2), and are consistent with the expectations from DFT studies where CO is bound to PtO_2_ species dispersed on anatase TiO_2_(101) surface. Further, Pt/TiO_2_-truncated bipyramid contains a similar amount of F ions as Pt/TiO_2_-nanosheet (F/Ti from XPS is 0.11 for Pt(0.25)/TiO_2_-truncated bipyramid and 0.26 for Pt(0.25)/TiO_2_-nanosheet, Supplementary Table 1). This indicates that F ions do not significantly affect the catalytic properties of Pt SAs in Pt/TiO_2_ SACs, and instead that the exposed surface TiO_2_ facets for each sample is what mainly controls the preferred Pt SA coordination environment and catalytic activity.

In conclusion, trends in CO oxidation activity and ${E}_{a}$ for Pt/TiO_2_-commercial, Pt/TiO_2_-nanosheet and Pt/TiO_2_-truncated bipyramid catalysts correlated to detailed structural characterization provide strong evidence that the reaction is catalyzed by Pt SAs dispersed on the (101) surface, whereas Pt SAs dispersed on the (001) surface have lower activity. This leads to the important conclusion that for Pt/TiO_2_-commercial, some Pt SAs were located in the TiO_2_ bulk under (001) surfaces and thus were not considered in previous structural or reactivity analyses. Therefore, our results emphasize that accurately assessing the structural distribution of Pt SA in SACs is critical for establishing structure-function relationships.

## Methods

### Synthesis of well-defined TiO_2_-nanosheet

The synthesis was conducted through a two-step hydrothermal route^50^. A specific amount of 1-butanol diluted titanium butoxide and hydrofluoric acid (48.0 wt.%) were mixed with the Ti to F molar ratio (Ti/F) of 1:2. The mixture was first added into a 50 mL Teflon-lined stainless-steel autoclave after being stirred for 1 hr at room temperature and then kept at 160 °C for 24 h. Following the reaction, the white precipitates were separated through centrifugation, and the supernatant was transferred into another Teflon-lined stainless-steel autoclave and kept at 210 °C for 4 h. The resulting white precipitate was washed several times using pure ethanol, pure acetone, and water, and then sonicated for 1 day. Finally, the sample was dried under vacuum at ambient temperature for 1 day.

### Synthesis of well-defined anatase TiO_2_-truncated bipyramid

A certain amount of 1-butanol diluted titanium butoxide and hydrofluoric acid (48.0 wt.%) were mixed with a Ti/F of 4:1. The mixture was added to a 50 mL Teflon-lined stainless-steel autoclave after being stirred for 1 h at room temperature and kept at 160 °C for 24 h. After the reaction, the white precipitates were separated through centrifugation, and the supernatant was transferred to another Teflon-lined stainless-steel autoclave and kept at 210 °C for 60 h. The resulting light blue precipitate was washed multiple times by pure ethanol, pure acetone, and water, and sonicated for 1 day. Finally, the sample were dried in vacuum at ambient temperature for 1 day.

### Distribution of Pt to TiO_2_ support

Pt was dispersed on TiO_2_ support by using a modified strong electrostatic adsorption (SEA) method^3,4^. SEA samples were prepared at a range of Pt weight loadings from 0.025 to 1 wt.%. Briefly, 0.5 g of TiO_2_ was dispersed in 50 mL of deionized H_2_O, and the pH of solution was adjusted to 12.2 by using NH_4_OH solution. Separately, the desired amount of tetraammineplatinum(II) nitrate (TAPN) was dissolved in deionized H_2_O, whose pH was also set at 12.2. Afterward, the TAPN solution was slowly injected into the TiO_2_ solution over 12.5 h, and the final solution was heated to 70 °C until completely dried. This ensures that the targeted amount of Pt is loaded on TiO_2_ support. The dried Pt/TiO_2_ samples were calcined in a tube furnace either at 300 °C (Pt/TiO_2_-nanosheet and Pt/TiO_2_-truncated bipyramid) or at 450 °C (Pt/TiO_2_-commercial) for 4 h by flowing dry air after ramping up the temperature at a rate of 10 °C/min. In addition, to check the structural change in TiO_2_ particles, the samples were reduced with 10% H_2_/Ar at 250 °C for 2 h after ramping up the temperature at a rate of 10 °C/min.

### Scanning transmission electron microscopy (STEM)

Regular HAADF-STEM characterization was applied by a JEOL Grand ARM 300CF microscope, which is equipped with a cold field emission gun (FEG) and double spherical aberration correctors. This microscope was operated at 300 kV, providing a spatial resolution of 63 pm. All HAADF-STEM images were acquired with a probe current of 23 pA, using a convergence semi-angle of 21 mrad and inner- and outer- collection angles of 79 and 180 mrad, respectively. The depth sectioning HAADF-STEM was performed using a Nion UltraSTEM200 microscope equipped with a cold FEG and a C_3_/C_5_ aberration corrector. This microscope was operated at 60 kV, providing a spatial resolution of 1 Å. The images were collected through different focal series using a larger convergence semi-angle of 38 mrad and inner- and outer-collection angles of 75 and 210 mrad.

### General characterization

XPS measurements were carried out using a Kratos AXIS-Supra photoelectron spectrometer, equipped with a monochromatic Al-Kα X-ray source. Two distinct spots on the sample were analyzed during experiments to ensure the consistency in peak intensities for binding energy scans of C 1*s*, Ti 2*p*, O 1*s*, Pt 4*f* and Pt 4*d*, and to verify the overall uniformity of the sample. All data were collected using a low X-ray energy (30 watts) to minimize the beam damage effects. To further validate the STEM observations, which indicated that Pt SAs were situated beneath the TiO_2_ nanosheet’s surface, the Pt(0.25)/TiO_2_-nanosheet sample was etched using an Ar ion beam composed of clusters of 1000 Ar atoms at 10 keV. This ion beam was rastered over an area of 2 mm × 2 mm for a duration of 10 s, resulting in the removal of the top surface of the TiO_2_ nanosheet. Pt 4*f* and 4*d* XPS spectra were compared before and after the etching procedure to show the differences in intensity and further infer the Pt location. X-ray powder diffraction (XRD) spectra were collected using a Mode 1 Smartlab diffractometer with Cu-Kα radiation (λ = 0.1542 nm) at a voltage of 40 kV and a current of 30 mA. The scanning-step size was 0.02° at a speed of 2.5 °/min. The Scherrer equation was used to estimate the average crystallite size. For surface area measurement, N_2_ adsorption-desorption isotherms were studied using a Micromeritics 3Flex Porosimeter at liquid N_2_ temperature. Prior to analysis, all samples were degassed under vacuum at 300 °C for 2 h. The specific surface area was calculated using the Brunauer-Emmett-Teller (BET) method.

### Fourier transform infrared (FTIR) spectroscopy

FTIR spectra were collected in a diffuse reflectance reaction chamber (Harrick Scientific) equipped with ZnSe windows, mounted inside a Praying Mantis diffuse reflectance adapter (Harrick Scientific), and coupled to a Thermo Scientific Nicolet iS10 FTIR spectrometer with a liquid-nitrogen-cooled HgCdTe (MCT) detector. The FTIR and Praying Mantis diffuse reflection accessory were purged with dry N_2_ while doing the experiments. In a typical experiment, the reactor was loaded with ~80 mg of 50 nm-sized γ-Al_2_O_3_ (Sigma Aldrich), followed by 15 mg of sample packed on top of the inert alumina. For the spectra collected after the oxidative pretreatment, samples were oxidized with 100% O_2_ at 300 °C for 1 h, cooled down to 25 °C under O_2_ and purged with Ar at 25 °C for 30 min before taking the background spectra. For the spectra collected after the reductive pretreatment, samples were reduced with 10% H_2_ at 250 °C for 1 h, purged with Ar at 250 °C for 30 min, and cooled down to 25 °C under Ar before taking the background spectra. 10% CO/Ar was flowed to the sample at 25 °C for 10 min. The flow rate was maintained at 50 mL/min. To estimate the CO-IR band intensities under the CO flow, the gas-phase CO signal was removed from the spectra by data post-processing. The *operando* CO-IR spectra were collected under the CO oxidation reaction conditions of 1% CO, 10% O_2_ and Ar balance at a total flow rate of 50 mL/min. Spectra were collected after running the reaction for 1 h at each temperature. The temperature was lowered from 300 to 25 °C so that surface reconstructions induced by the reaction feed did not affect the spectral interpretations.

### CO oxidation reactivity measurements

CO oxidation was performed in a kinetically controlled regime (CO conversion <15%)^4^. To confirm that the activity differences between catalysts are due to kinetic effects, we performed the CO oxidation reaction at different space velocities by varying the amount of catalyst tested (Supplementary Fig. 22). The measured rates are similar to each other over a wide space velocity range, suggesting that the rates measured here were obtained under the kinetically controlled region. For activity measurement, the catalysts were oxidized at 300 °C for 2 h while flowing 10% O_2_/Ar. Afterwards, a reaction feed consisting of 1% CO, 10% O_2_ and Ar balance was flowed to the catalyst while varying the temperature from 300 to 250 °C for Pt/TiO_2_-commercial and Pt/TiO_2_-nanosheet, or from 290 to 250 °C for Pt/TiO_2_-truncated bipyramid. CO oxidation reactions were run for 1 h at each temperature, and in all cases the steady-state activity was reached within 30 min. The flow rate was always maintained at 50 mL/min, and the effluent gas was analysed using online mass spectrometry (Hiden Analytical).

### XPS fitting method

To confirm the location of Pt SAs in both TiO_2_-nanosheet and -truncated bipyramid, a comprehensive XPS analysis was conducted. Given that Pt 4 *f* signal overlaps with Ti 3 *s* energy loss, we collected XPS signals from samples with and without Pt to accurately assess the intensity and location of Ti 3 *s* energy loss peak and minimize its influence on the Pt 4 *f* signal. The detailed procedure is as follows: (1) Spectra calibration: All the spectra were initially calibrated using the position of C 1 *s* peak. (2) Ti 3 *s* energy loss peak fitting: The Ti 3 *s* energy loss curves in TiO_2_-nanosheet and -truncated bipyramid without Pt were first normalized to the range of 0 to 1, followed by assigning a Shirley-type background. The Ti 3 *s* energy loss peaks were then fitted in TiO_2_-nanosheet (peak center = 75.3 eV, FWHM = 3.0, peak area = 1.5) and -truncated bipyramid (peak center = 75.6 eV, FWHM = 3.1, peak area = 1.5), respectively. (3) Pt 4 *f* peak fitting: A Shirley-type background curve was assigned to Pt(0.25)/TiO_2_-nanosheet and Pt(0.25)/TiO_2_-truncated bipyramid in the Pt 4 *f* and Ti 3 *s* energy loss overlapping region. Ti 3 *s* energy loss peaks in Pt(0.25)/TiO_2_-nanosheet and Pt(0.25)/TiO_2_-truncated bipyramid were then fitted and normalized using the above constraint parameters (peak center, FWHM, peak area). The remaining signal in this region were fitted to Pt 4 *f*, with a fixed Pt 4*f*_5/2_ to Pt 4*f*_7/2_ ratio of 0.71, Pt 4*f*_7/2_ − 4*f*_5/2_ splitting of 3.2 eV, and FWHM of Pt 4*f*_7/2_ and Pt 4*f*_5/2_ at 2.2 eV and 3.1 eV, respectively. (4) Pt_4*f*_/Ti_3*s*_ calculation: The relative area fit ratio of Pt 4*f* to Ti 3*s* energy loss was then calculated to infer the differences in Pt SA location.

### Supplementary information

Supplementary Information

Inventory of Supporting Information

## Data Availability

The authors declare that the data that support the findings of this study are available within the article and its Supplementary Information files. All other relevant data are available from the corresponding authors upon request.
