# Supplementary material for: Distribution of Pt single atom coordination environments on anatase TiO2 supports controls reactivity
Source: Nat Commun. 2024 Feb 2;15:998. doi: 10.1038/s41467-024-45367-z (PMC10837418; doi:10.1038/s41467-024-45367-z)
Supplement: Supplementary file 1 — Supplementary Information [file 41467_2024_45367_MOESM1_ESM.pdf]

Supplementary Information

Distribution of Pt Single Atom Coordination Environments on Anatase TiO_2 Supports Controls Reactivity

Wenjie Zang[1*], Jaeha Lee[2*], Peter Tieu[3], Xingxu Yan[1], George W. Graham[1,4], Ich C. Tran[5], Peikui Wang[6], Phillip Christopher[2], Xiaoqing Pan[1,5,7]

1 Department of Materials Science and Engineering, University of California, Irvine, CA 92697, USA

2 Department of Chemical Engineering, University of California, Santa Barbara, CA 93106, USA

3 Department of Chemistry, University of California, Irvine, CA 92697, USA

4 Department of Materials Science and Engineering, University of Michigan, Ann Arbor, MI 48109, USA

5 Irvine Materials Research Institute, University of California, Irvine, CA 92697, USA

6 Department of Chemistry, University of Sherbrooke, Sherbrooke, QC J1K 2R1, Canada

7 Department of Physics and Astronomy, University of California, Irvine, CA 92697, USA

*These authors contributed equally to this work. Correspondence and requests for materials should be addressed to P.C. (email: pchristopher@ucsb.edu) or to X.P. (email: xiaoqinp@uci.edu).

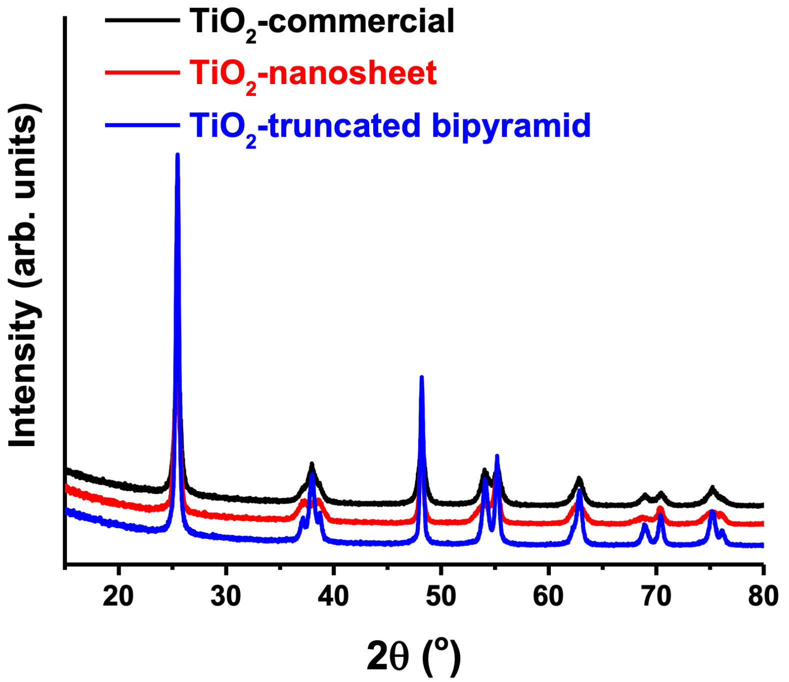

Supplementary Figure 1. XRD patterns of TiO_2-commercial, TiO_2-nanosheet, and TiO_2-truncated bipyramid supports. XRD patterns show that these TiO_2 samples with different morphologies are in an anatase phase.

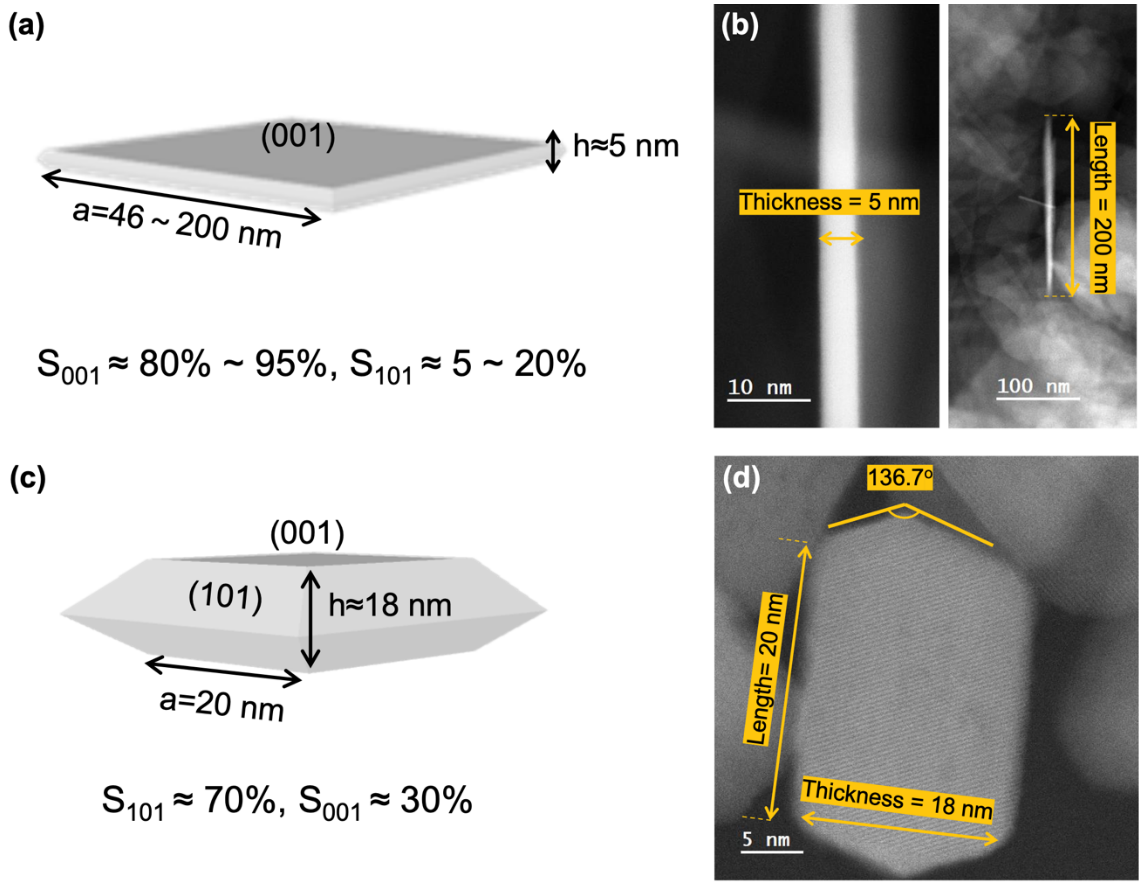

Supplementary Figure 2. The relative ratio of (101) and (001) surfaces exposed on (a-b) TiO_2-nanosheet and (c-d) TiO_2-truncated bipyramid. The relative ratio of (101) and (001) (marked as S_{101} and S_{001}, respectively) were calculated by dividing the surface area of (101) and (001) by total surface area of TiO_2-nanosheet/-truncated bipyramid. The surface area of each facet was determined by analysing multiple STEM images and measuring the length and width of each facet using Digital Micrograph software((b) and (d)).

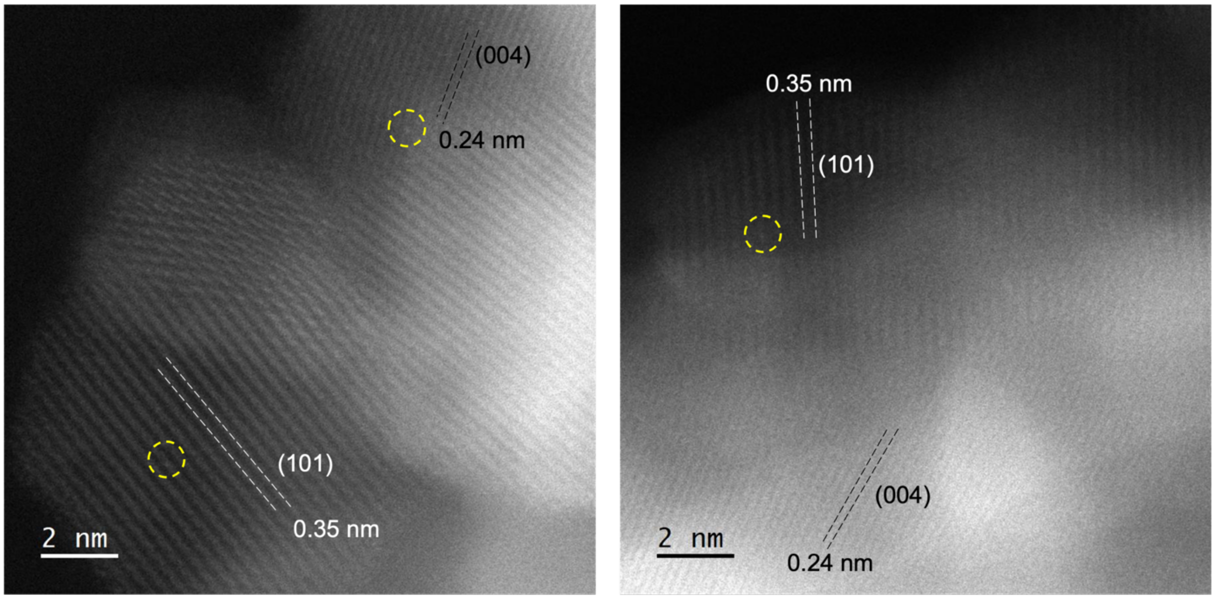

Supplementary Figure 3. HAADF STEM images of Pt(0.025)/TiO_2-commercial showing the co-existence of (101) and (001) surfaces. The lattice spacing were measured by Digital Micrograph software and marked by white/black dotted line, and Pt SAs were marked by yellow dotted circles.

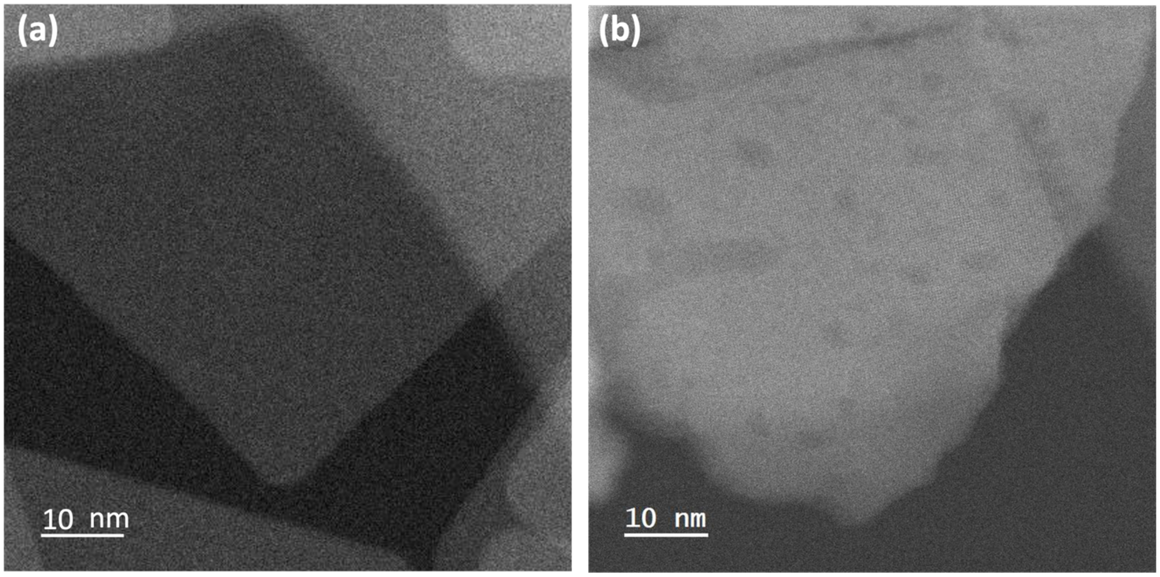

Supplementary Figure 4. The structure of TiO_2-nanosheet after oxidation conditions, specifically at (a) 300 ^oC and (b) 450 ^oC. The shape of nanosheet structure maintains well after annealing at 300 ^oC, however noticeable deformation occurs at the edges after annealing at 450 ^oC.

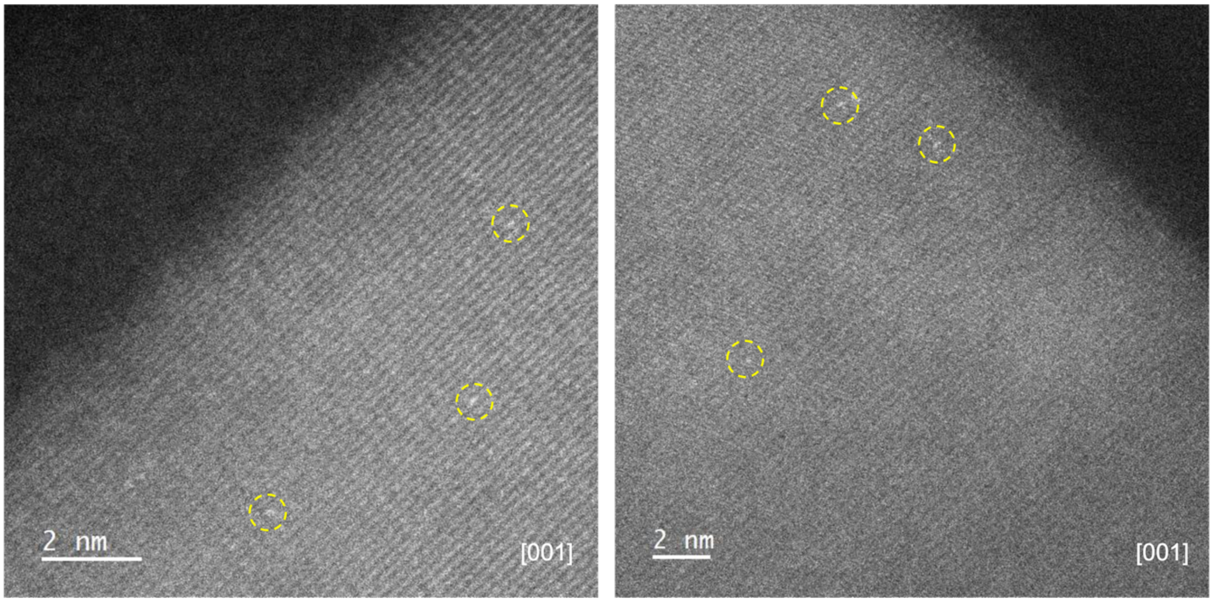

Supplementary Figure 5. HAADF STEM images showing the morphology of Pt(0.05)/TiO_2-nanosheet. All the Pt exist as single atoms (SAs, marked by yellow dotted circles) on the specifically oriented (001) surface.

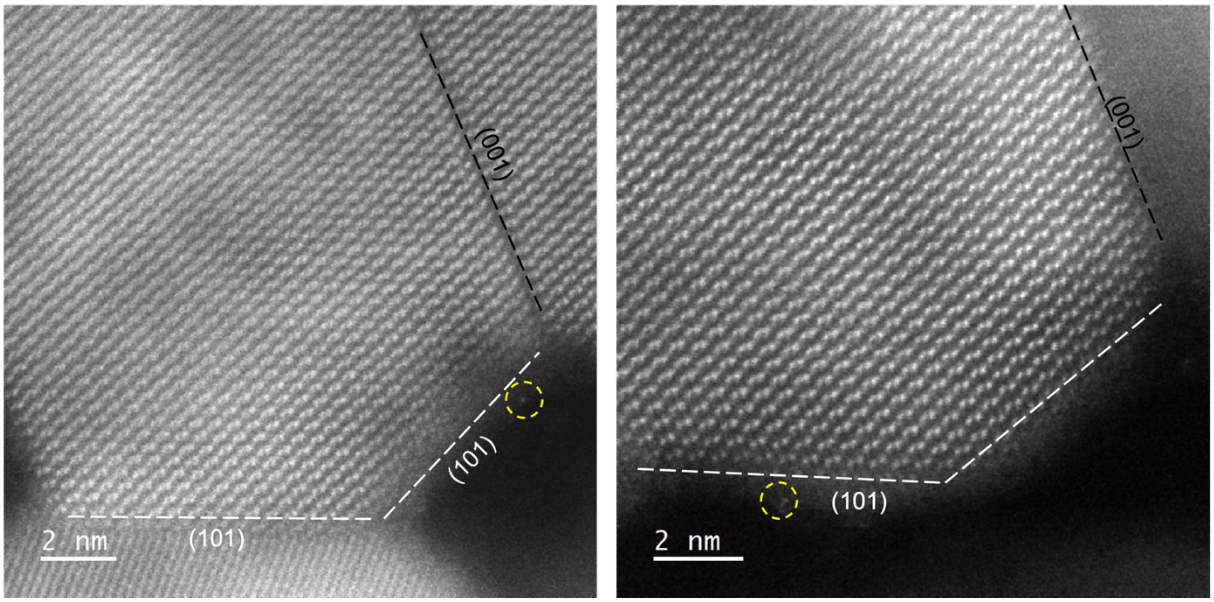

Supplementary Figure 6. HAADF STEM images showing the morphology of Pt(0.05)/TiO_2-truncated bipyramid. All the Pt exist as SAs (marked by yellow dotted circles) on the specifically oriented (101) surface.

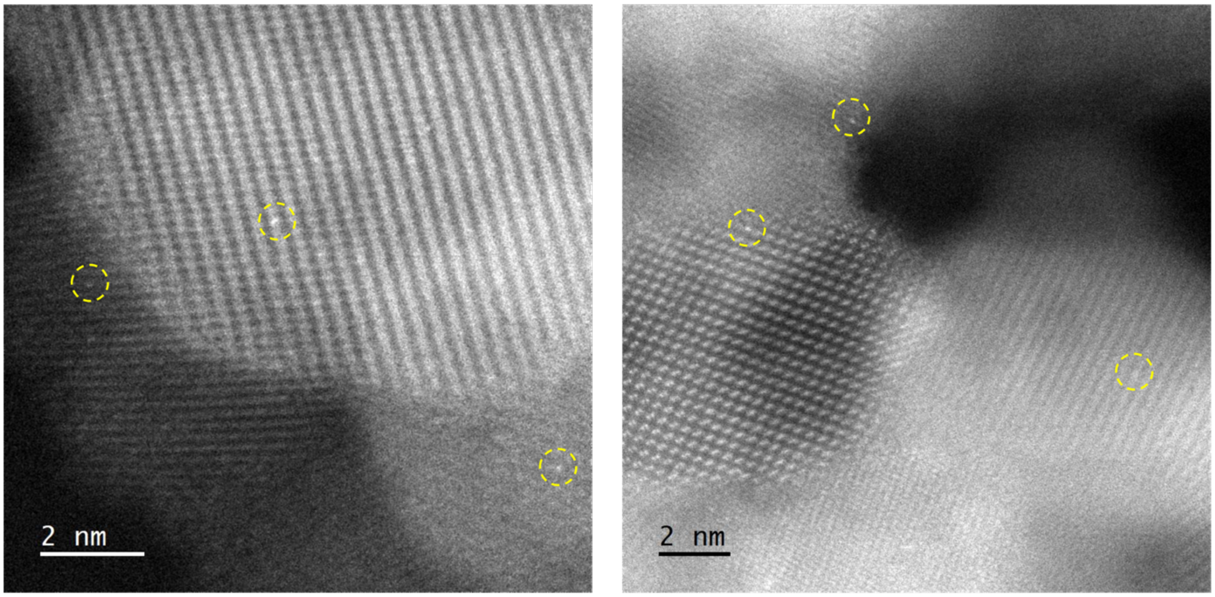

Supplementary Figure 7. HAADF STEM images of Pt(0.025)/TiO_2-commercial. Pt SAs were marked by yellow dotted circles.

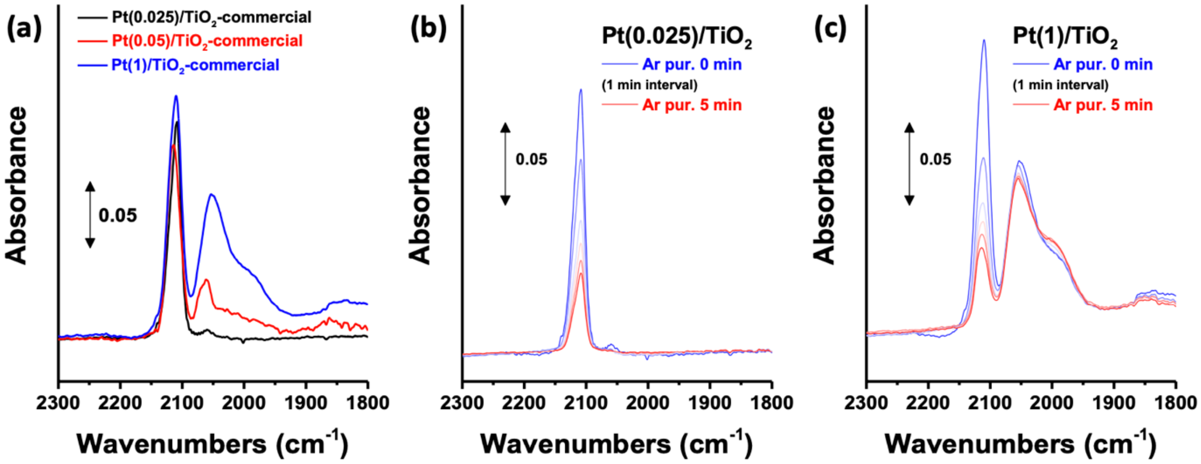

Supplementary Figure 8. CO-IR spectra of Pt/TiO_2-commercial with different Pt loadings. (a) CO-IR spectra of Pt/TiO_2-commercial with Pt loadings of 0.025, 0.05, and 1 wt.%. Spectra were collected after flowing 10% CO at 35 ºC for 10 min, and the gas-phase CO signal was manually removed. (b-c) CO-IR spectra of (b) Pt(0.025)/TiO_2-commercial and (c) Pt(1)/TiO_2-commercial. The spectra were collected while purging with Ar after flowing 10% CO for 10 min. Before flowing CO, samples were reduced with 10% H_2 at 250 ºC for 1 hr, followed by purging with Ar at 250 ºC for 30 min.

Supplementary Discussion I, Summary of previous literature on the validation of near 100% Pt dispersion on Pt(0.025)/TiO_2-commercial sample.

It was demonstrated in our prior studies that Pt can be exclusively atomically dispersed on TiO_2 supports by using small oxide nanoparticles as supports and depositing less than one Pt atom per particle.[1,2] In this study, TiO_2-commercial represents anatase TiO_2 particles with a diameter of ~5 nm (surface area, 290 m^2/g, US Research Nanomaterials), and 0.025 wt.% of Pt corresponds to ~0.2 Pt atom per TiO_2 particle.

Supplementary Figure 8a shows CO-IR spectra of Pt/TiO_2-commercial with different Pt loadings (0.025, 0.05 and 1 wt.%) collected after an *in-situ* reduction with 10% H_2 at 250 °C for 1 hr. At a Pt loading of 0.025 wt.%, the predominant CO stretching band is observed at ~ 2112 cm^{-1} which was attributed to CO bound to Pt SA.[1,2] In addition, another broader CO stretching band is observed at the lower wavenumbers, in the range of 1900-2100 cm^{-1}, at the higher Pt loading, which was attributed to CO bound to Pt NP.[2] When purged with Ar at 25 °C, the CO-IR band intensity at ~ 2112 cm^{-1} decreased rapidly (Supplementary Figure 8b-c), whereas the intensity of the CO-IR band in the range of 1900-2100 cm^{-1} did not (Supplementary Figure 8c). This agrees with previous reports that CO binds weakly to Pt SA but strongly to Pt NP. Supplementary Figure 8 shows that Pt is mostly atomically dispersed on TiO_2-commercial at a Pt loading of 0.025 wt.%. Consistent with our conclusions from CO-IR spectra, only Pt SAs could be found in the STEM images of Pt(0.025)/TiO_2-commercial (Supplementary Figure 7).

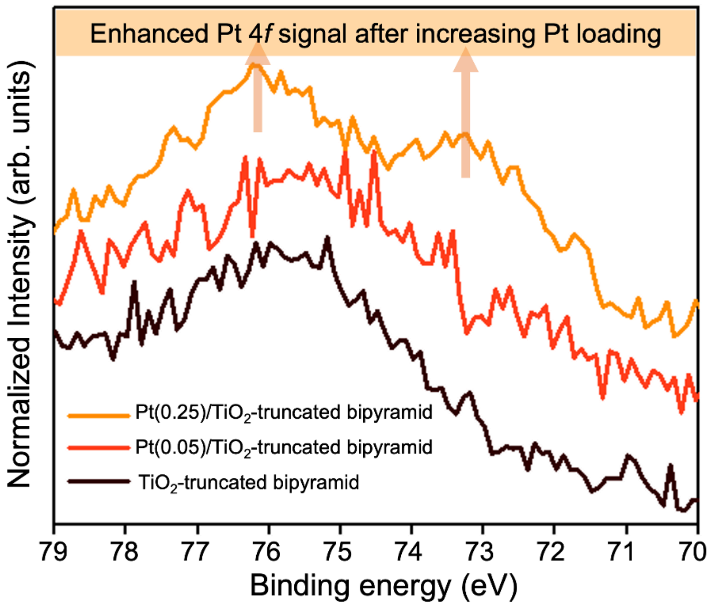

Supplementary Figure 9. XPS spectra of TiO_2-trucated bipyramid with different Pt loading, 0 wt.%, 0.05 wt.%, and 0.25 wt.%. The broad Ti 3*s* energy loss peak from pure TiO_2 supports and the shorter mean free path of Pt than Ti hinder the observation of Pt 4*f* XPS signal at a low Pt content of 0.05 wt.%. This signal was enhanced after increasing the Pt content to 0.25 wt.%. The morphology of Pt(0.05)/TiO_2-truncated bipyramid and Pt(0.25)/TiO_2-truncated bipyramid are shown in Supplementary Figure 6 and Supplementary Figure 14, respectively.

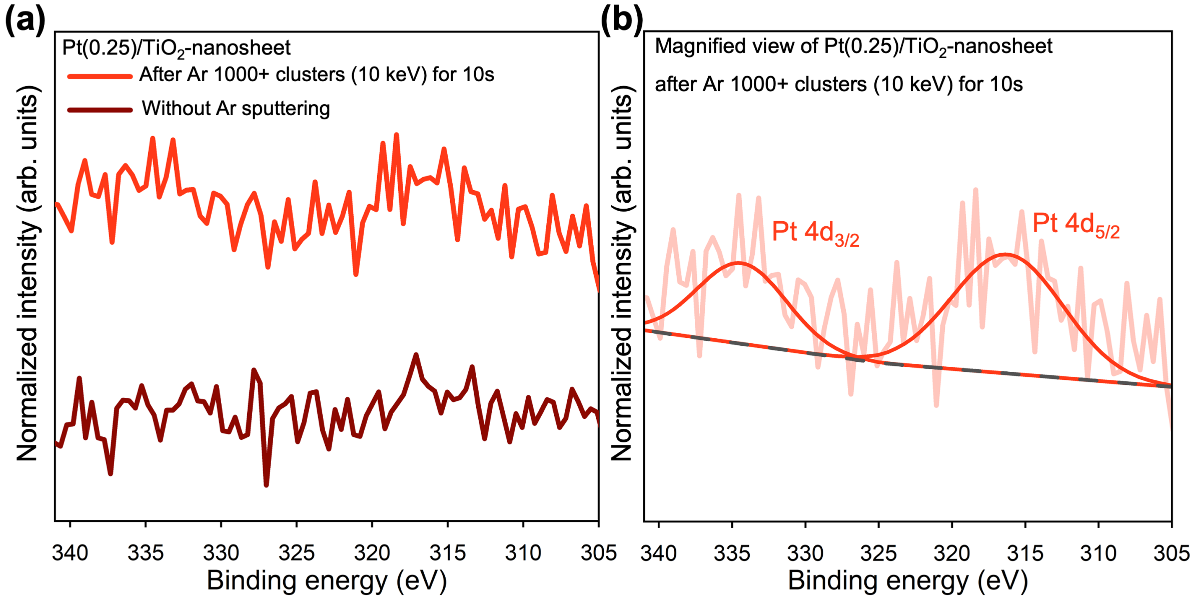

Supplementary Figure 10. Pt 4*d* XPS spectra of Pt(0.25)/TiO_2-nanosheet before and after Ar sputtering. (a) Spectra before and after sputtering with Ar_{1000} clusters at 10 keV for 10s. (b) Magnified view of Pt 4*d* spectrum after sputtering and corresponding fitted spectrum. The increase in the Pt signal after Ar sputtering compared to its initial level indicates that a large fraction of Pt is located in the bulk of TiO_2-nanosheet.

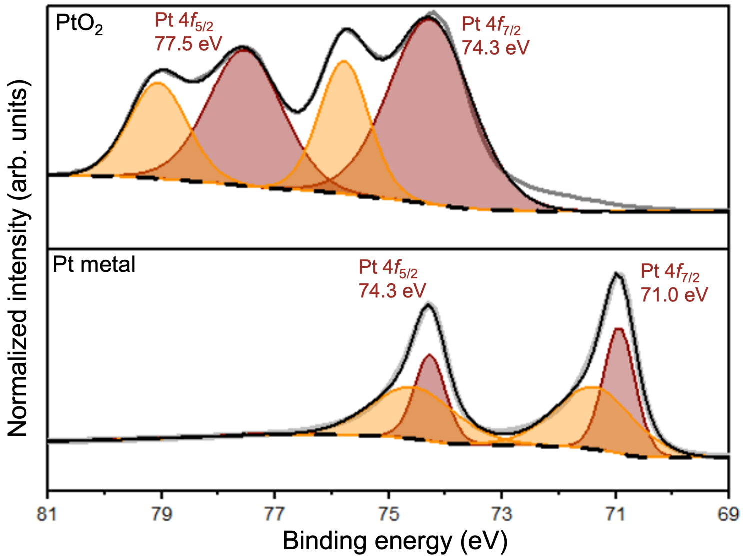

Supplementary Figure 11. Pt 4*f* XPS spectra of metallic Pt and PtO_2 with Pt valence states of 0 and +4. It should be noted that PtO_2 is sensitive to low energy X-rays (30 watts), leading to a shift in XPS peak towards the higher energy, as shown by the fitted orange peaks in PtO_2. However, despite this sensitivity, a large amount of PtO_2 still remains after X-ray exposure, as evidenced by the fitted red peaks.

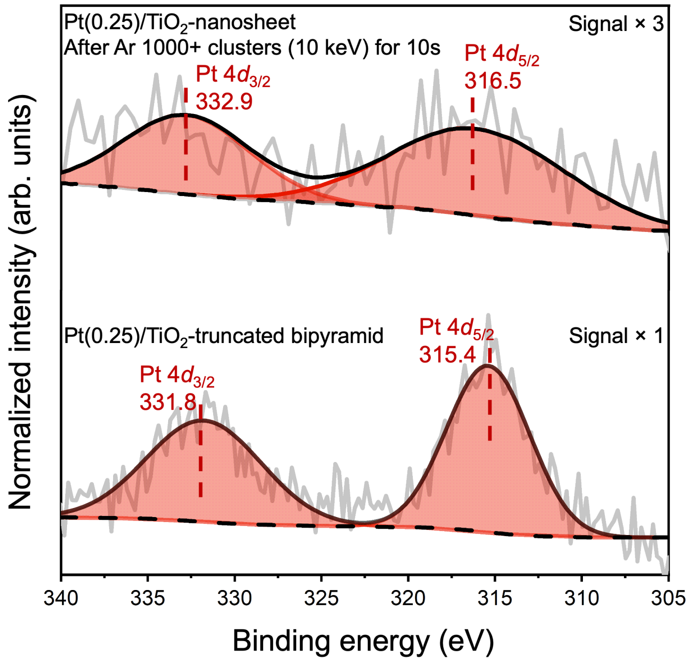

Supplementary Figure 12. Normalized Pt 4*d* XPS spectra of Pt(0.25)/TiO_2-truncated bipyramid and Pt(0.25)/TiO_2-nanosheet after sputtering with Ar_{1000} clusters. The signals of the original and fitted Pt 4d spectra for Pt(0.25)/TiO_2-nanosheet after Ar sputtering have been magnified for threefold to enhance visibility and make a comparison with Pt(0.25)/TiO_2-truncated bipyramid.

Besides, it should be noted that the XPS signals from Pt 4*d* overlap with those from C 1*s* which display a broad peak centered at 307 eV. To mitigate the impact of C interferences, the XPS spectra of Pt(0.25)/TiO_2-truncated bipyramid and -nanosheet were subtracted by the XPS spectra of Pt-free TiO_2-nanosheet and TiO_2-truncated bipyramid.

Supplementary Discussion II, Estimation of the Pt oxidation state using XPS spectra.

Based on the experimental XPS spectra of Pt metal and PtO_2 (IV) shown in Supplementary Figure 11, along with the reference Pt 4*f* binding energy in Pt metal foil, PtO (II), and PtO_2 (IV) as mentioned in the literature,[3-5] the Pt $4f_{7/2}$ binding energies for Pt^0 and Pt^{2+} and Pt^{4+} are determined to be 71.0, 72.4, 74.3 eV, respectively. These values have been marked as three blue dots in the figure displayed below. It is observed that these three dots show a linear relationship between Pt oxidation state and Pt $4f_{7/2}$ binding energy, represented in a blue dot line, indicating that Pt $4f_{7/2}$ binding energy increases linearly with the rise of Pt valence state.

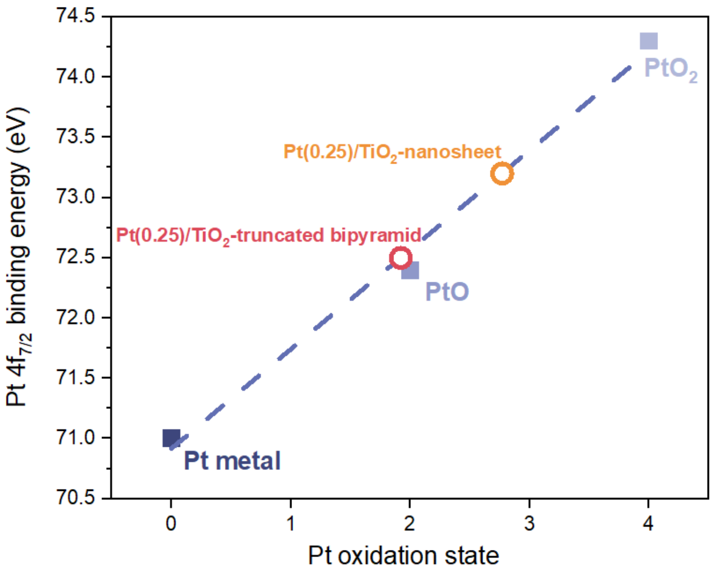

In Figure 2, the Pt $4f_{7/2}$ binding energies of Pt(0.25)/TiO_2-truncated bipyramid and Pt(0.25)/TiO_2-nanosheet are reported as 72.5 eV and 73.2 eV. By applying these binding energies to the previously established linear fitted curve, it is determined that the corresponding Pt oxidation states for Pt(0.25)/TiO_2-truncated bipyramid and Pt(0.25)/TiO_2-nanosheet are approximately +1.9 and +2.8, respectively. The higher Pt valence state of Pt SAs in Pt(0.25)/TiO_2-nanosheet compared to Pt(0.25)/TiO_2-truncated bipyramid suggests that Pt SAs located at the subsurface of the TiO_2(001) in the nanosheet are more cationic and more coordinated with the oxygen atoms within the TiO_2 lattice, as compared to those on the TiO_2(101) surface.

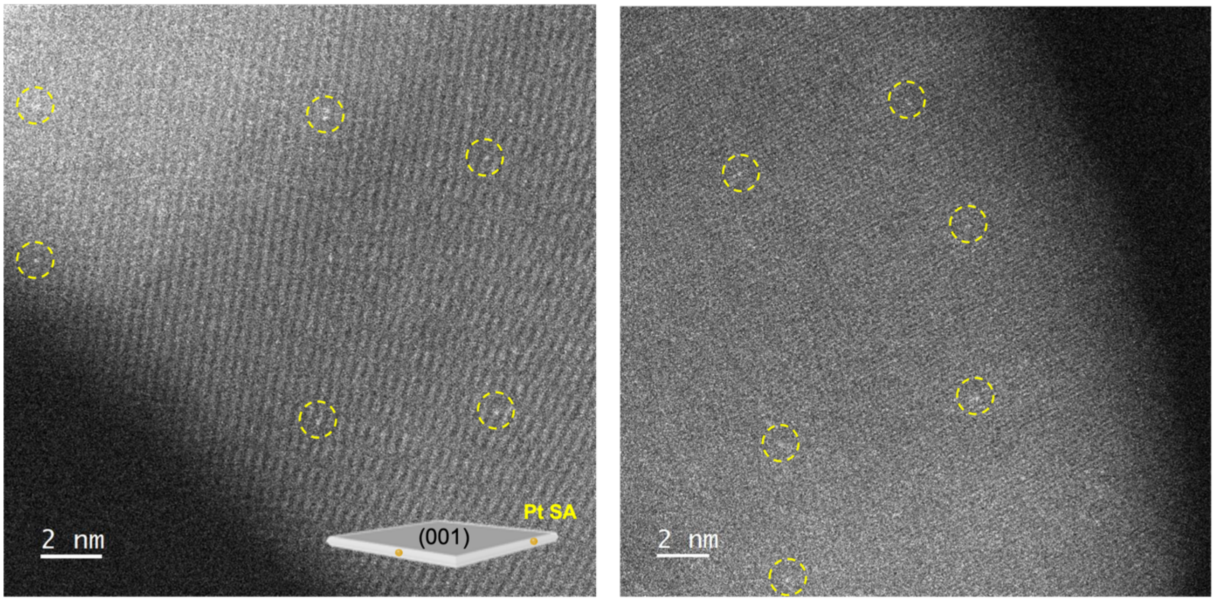

Supplementary Figure 13. HAADF STEM images of Pt(0.25)/TiO_2-nanosheet. Abundant Pt SAs are found (marked by yellow dotted circles) on the specifically oriented (001) surface. The observed number density of Pt SA is estimated to be ~0.03 atom/nm^2. This value is lower than the nominal surface coverage of 0.14 atom/nm^2, as one HAADF STEM image can only detect Pt SAs that exist in TiO_2 within the depth of field, and may not capture all Pt SAs throughout the entire thickness of TiO_2.

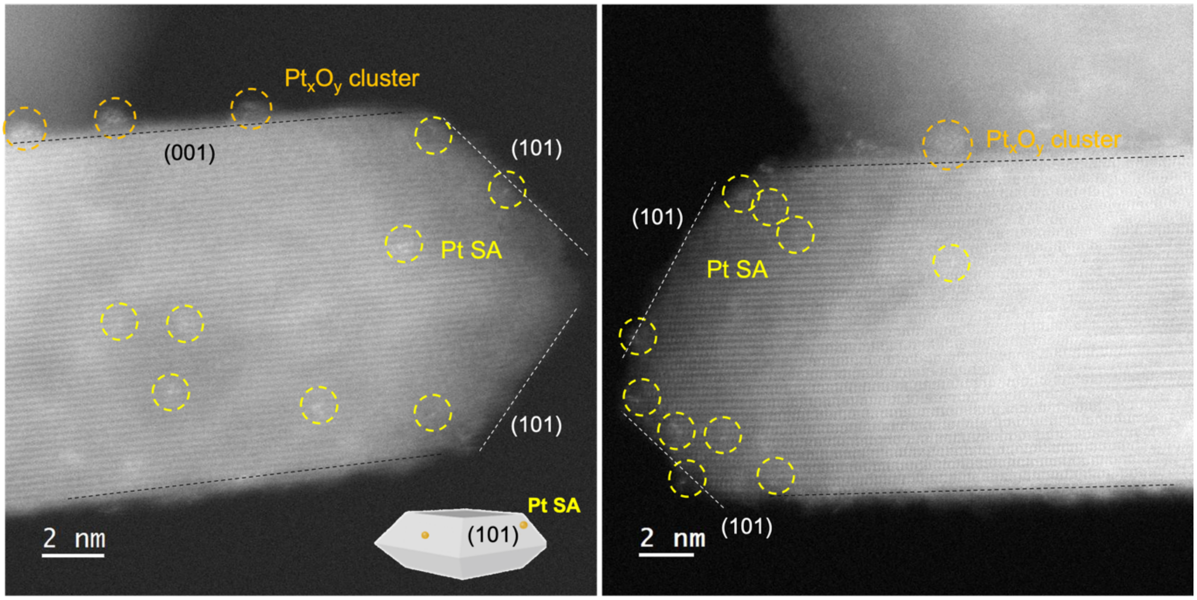

Supplementary Figure 14. HAADF STEM images of Pt(0.25)/TiO_2-truncated bipyramid oxidized at 300 °C. Most Pt are highly dispersed as SAs on (101) surfaces, as marked by yellow dotted circles, and a small amount of Pt_xO_y clusters is found on the (001) surface and marked by orange dotted circles. A quantitative assessment of Pt SAs indicates an observed density of around 0.02 atom/nm^2, which is lower than the nominal surface coverage of 0.13 atom/nm^2. This discrepancy can be attributed to the presence of Pt_xO_y clusters, and more importantly, the limited depth of focus, as not all Pt SAs on the TiO_2 (101) surface can be clearly observed with high contrast in a given image under the zone axis of [010].

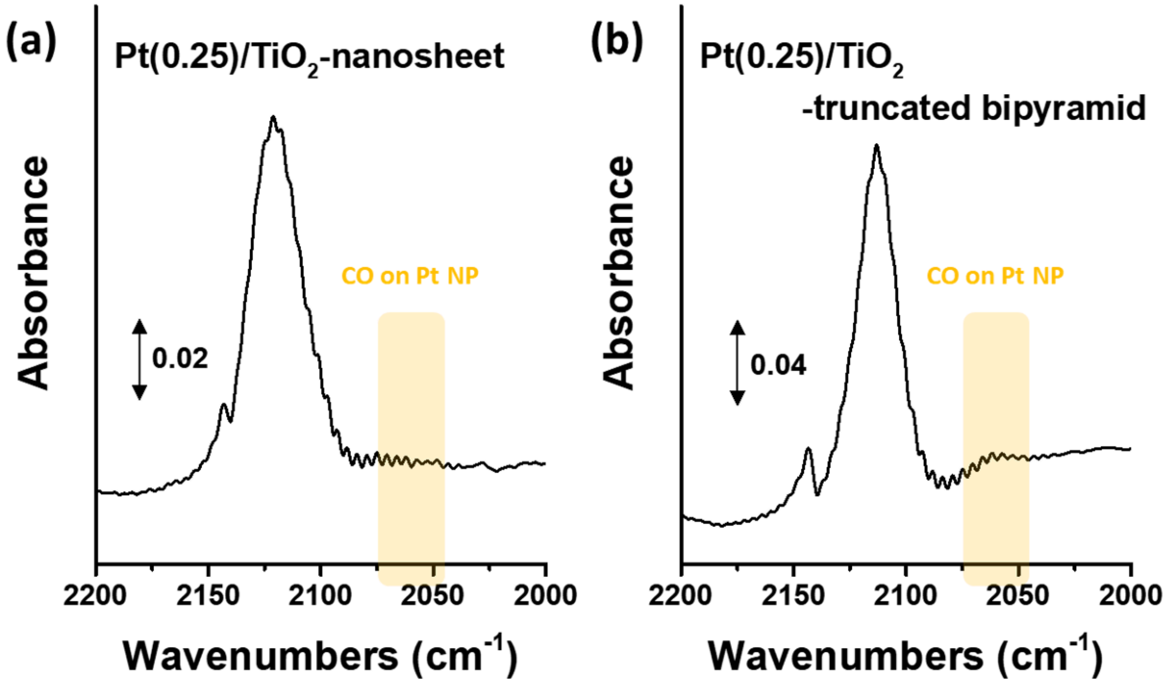

Supplementary Figure 15. IR spectra of (a) Pt(0.25)/TiO_2-nanosheet and (b) Pt(0.25)/TiO_2-truncated bipyramid collected after flowing 10% CO at 35 °C for 10 min. Gas-phase CO signal was manually removed. Here, samples were pretreated with 10% H_2 at 250 °C for 1 hr and purged with Ar at 250 °C for 30 min.

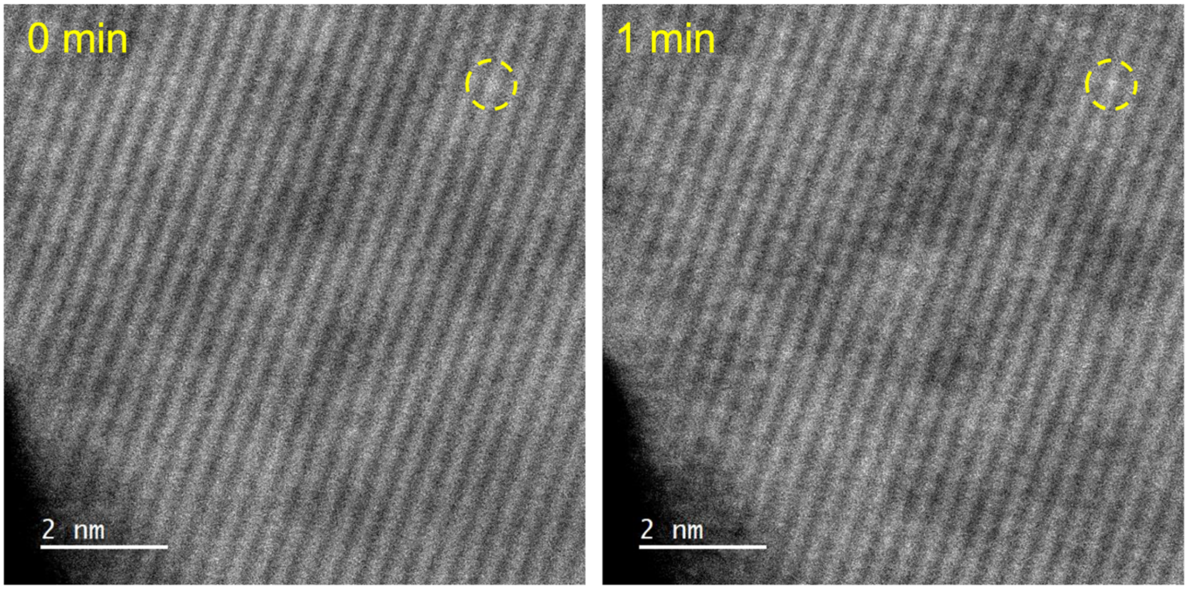

Supplementary Figure 16. HAADF STEM images of the same region captured before and after a 1-min period of continuous irradiation. The images demonstrate that there is negligible electron beam influence on the location of Pt SAs (highlighted with the yellow dotted circle) and the morphology of the TiO_2-nanosheet support. This observation suggests that the short-time image acquisition process does not significantly impact the mobility of Pt SAs or induce any notable damage to the TiO_2-nanosheet support.

Supplementary Table 1. Atomic ratios of Ti, F and O atoms. Atomic ratios of Ti, F, and O in (1) TiO_2-nanosheet, (2) Pt(0.25)/TiO_2-nanosheet, (3) Pt(0.25)/TiO_2-nanosheet after reduction with H_2, and (4) Pt(0.25)/TiO_2-truncated bipyramid are estimated from XPS spectra. (1), (2) and (4) samples were oxidized with air at 300 °C before collecting the spectra.

No.	Sample	Ti	F	O
1	TiO_2-nanosheet	1.00	0.35	2.29
2	Pt(0.25)/TiO_2-nanosheet	1.00	0.26	2.54
3	Pt(0.25)/TiO_2-nanosheet after reduction under 10% H_2/Ar at 250 °C for 2 hours	1.00	0.18	2.40
4	Pt(0.25)/TiO_2-truncated bipyramid	1.00	0.11	2.21

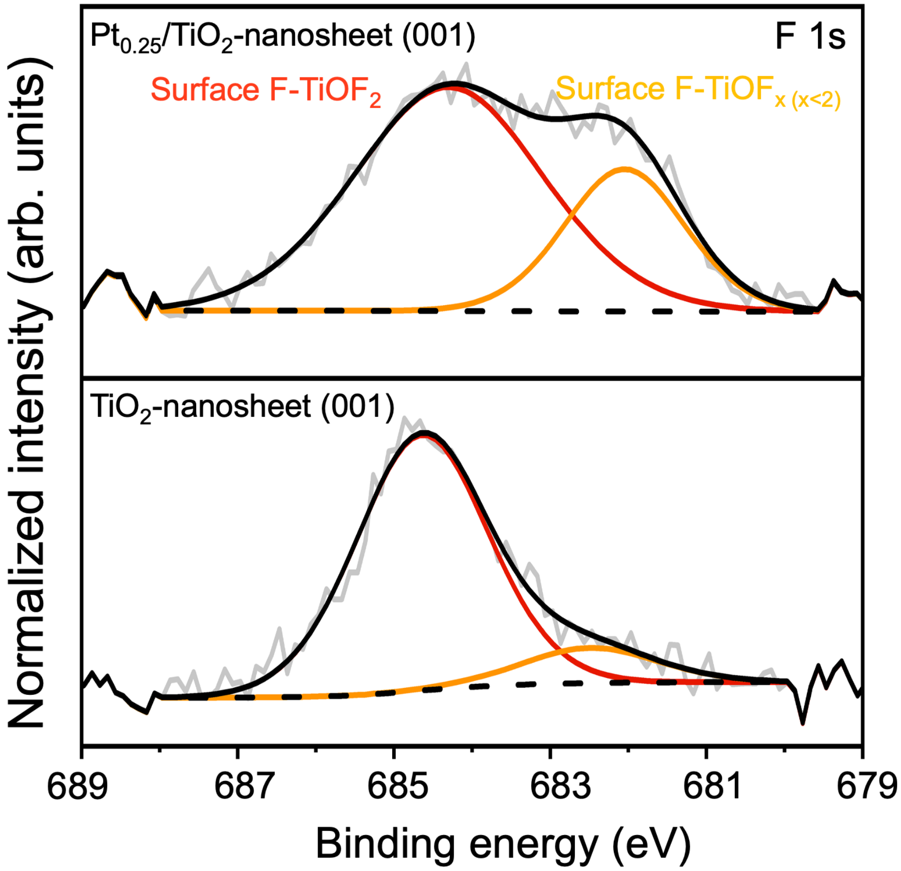

Supplementary Figure 17. F 1*s* XPS spectra of TiO_2-nanosheet with and without Pt. The peak located at 684.0-684.3 eV corresponds to the presence of F on the TiO_2 surface with $TiOF_2$ or Ti-F bonding. Another peak located at 682.0 eV represents the presence of surface F bonded on TiO_2 surface as $TiOF_x$ (x<2).

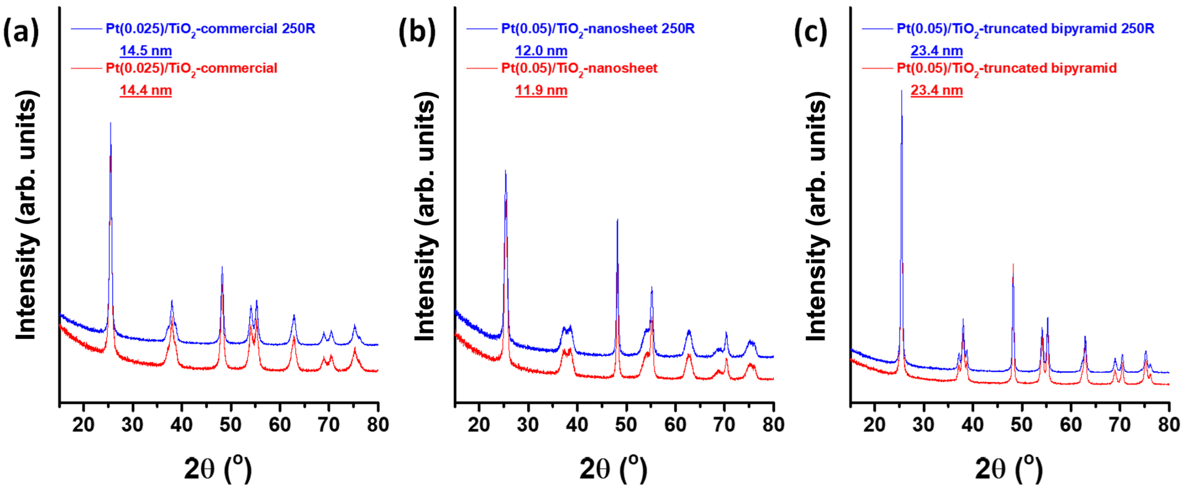

Supplementary Figure 18. XRD patterns of (a) Pt(0.025)/TiO_2-commercial, (b) Pt(0.05)/TiO_2-nanosheet and (c) Pt(0.05)/TiO_2-truncated bipyramid samples before and after reduction with 10% H_2 at 250 °C for 1 hr. The average crystalline size of TiO_2 is also calculated by using the Scherrer equation to the (101) diffraction peak near 25 °. Note that the shape factor (K) in Scherrer equation should be different for differently shaped TiO_2 particles. But it was set at 0.9 for all samples for comparison purposes.

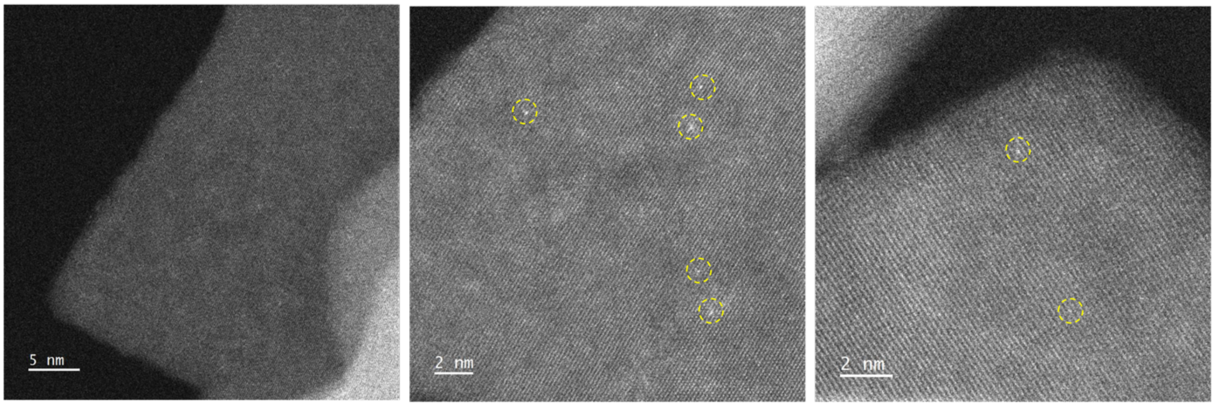

Supplementary Figure 19. Representative HAADF STEM images of Pt(0.25)/TiO_2-nanosheet after reduction with 10% H_2 at 250 °C for 1 hr. We could only observe Pt SAs, which are marked with yellow dotted circles. The estimated Pt SA surface density is ~0.025 atom/nm^2, comparable to the value estimated before the reduction treatment (Supplementary Figure 13). CO-IR spectra in Supplementary Figure 15a also support the conclusions from TEM images, as the band intensity from CO bound to Pt clusters is under the detection limit. That is, the concentration of Pt clusters falls below the detection limits of both TEM and CO-IR spectroscopy.

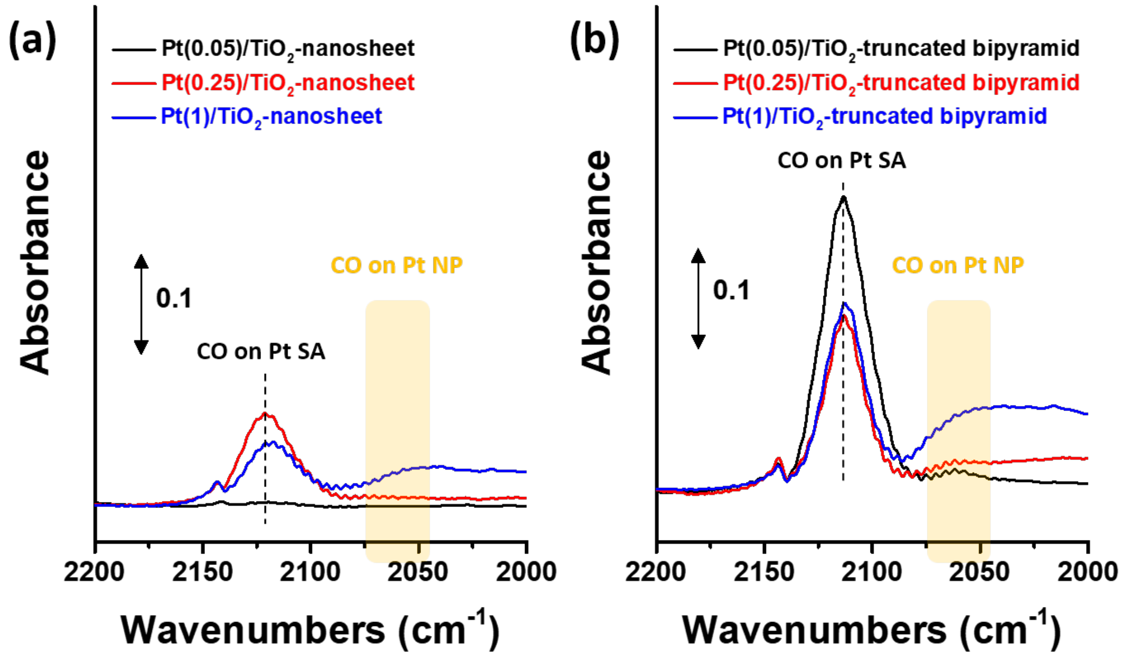

Supplementary Figure 20. CO-IR spectra of (a) Pt/TiO_2-nanosheet and (b) Pt/TiO_2-truncated bipyramid with different Pt loading (0.05, 0.25 and 1 wt.%). Spectra were collected after flowing 10% CO at 35 °C for 10 min, and the gas-phase CO signal was manually removed from the spectra. Before flowing CO, samples were reduced with 10% H_2 at 250 °C for 1 hr, followed by purging with Ar at 250 °C for 30 min.

Supplementary Table 2. Summary of the FWHM and the centroid of the IR band from CO bound to Pt SA dispersed on anatase TiO_2 support from our work and those from literature.

Sample	Peak center of CO on Pt SA (cm^{-1})	FWHM in A unit (cm^{-1})	FWHM in KM unit (cm^{-1})	Description	Reference
Pt(0.025)/TiO_2-US nano (commercial)	2109	20	13	Reduced with 10% H_2 at 250 °C	*This work*
Pt(0.05)/TiO_2-truncated bipyramid	2113	25	17	Reduced with 10% H_2 at 250 °C	*This work*
Pt(0.05)/TiO_2-US nano	2112	-	6-10	Reduced with 10% H_2 at 250 °C	*2*
Pt(0.025)/TiO_2-US nano	2112	11	-	Reduced with 10% H_2 at 250 °C	*1*
Pt(1)/TiO_2-US nano	2118	~13 (unit not specified)		Reduced with 10% H_2 at 250 °C	*6*
Pt(0.22)/TiO_2(101) surface	2090	~18 (unit not specified)		Treated with N_2 at 300 °C	*7*

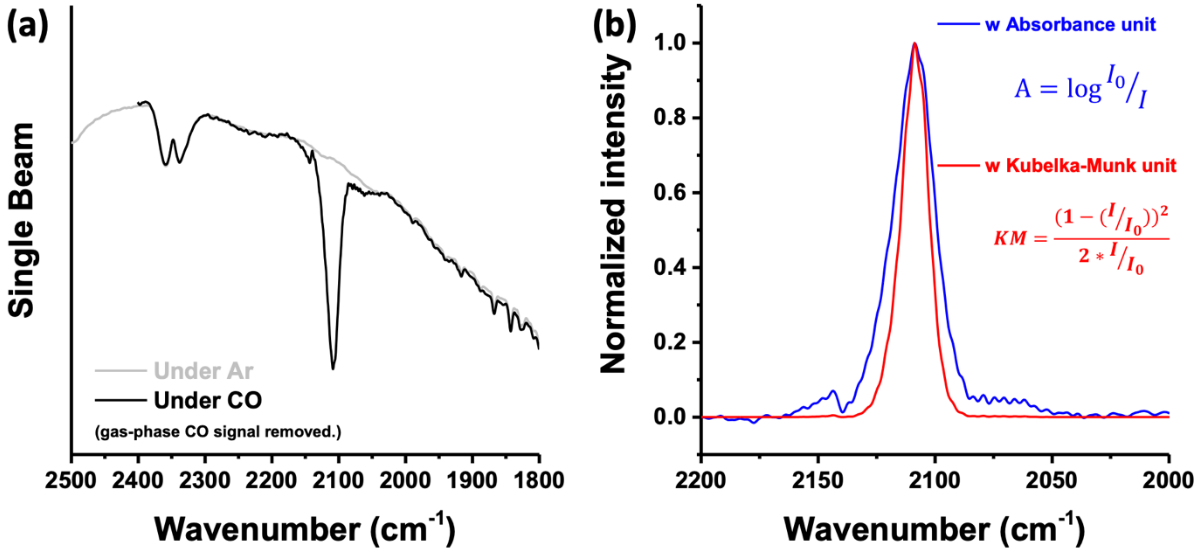

Supplementary Figure 21. Full-width at half maximum (FWHM) analysis using different units. (a) IR spectra of Pt(0.025)/TiO_2-commercial in Single Beam unit collected under Ar at 25 ºC and after flowing 10% CO at 25 ºC for 10 min. The gas-phase CO signal was manually removed from the spectra. Before flowing CO, samples were reduced with 10% H_2 at 250 ºC for 1 hr, followed by purging with Ar at 250 ºC for 30 min. (b) Comparison of normalized IR spectra in Absorbance and Kubelka-Munk units obtained from the spectra in (a), taking the one collected under Ar as background.

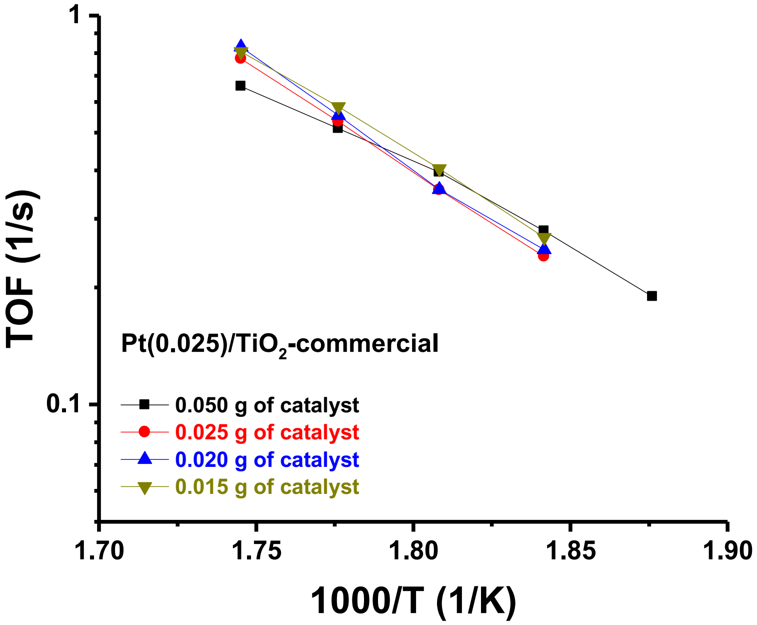

Supplementary Figure 22. Dilution study for Pt(0.025)/TiO_2-commercial. Different amount of catalyst was diluted with 0.3 g of SiO_2, and TOF was estimated at a CO conversion < 15%. The TOF measured at different space velocities were similar, indicating that the rates measured here were taken under kinetic control.

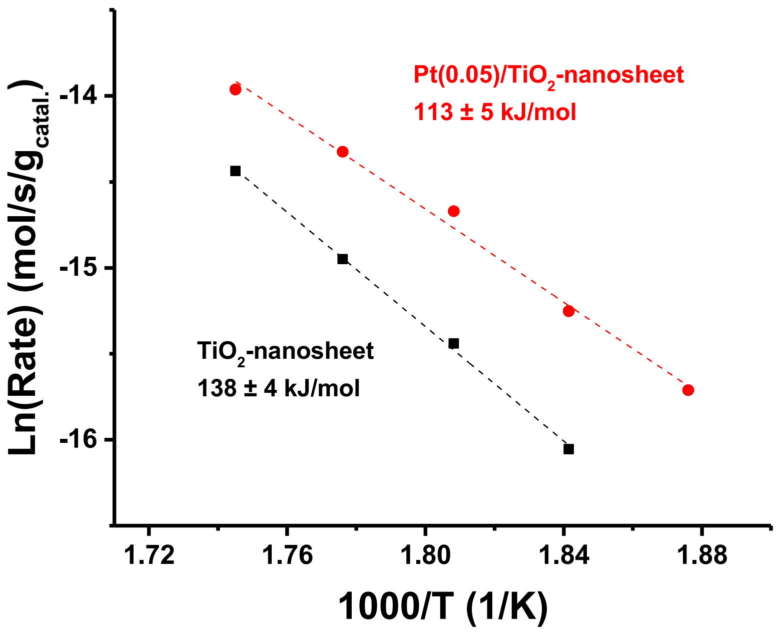

Supplementary Figure 23. The Arrhenius plots of TiO_2-nanosheet and Pt(0.05)/TiO_2-nanosheet. These plots show the temperature dependence of the per-catalyst rate (mol/s/$g_{catal.}$) for CO oxidation reaction on TiO_2-nanosheet and Pt(0.05)/TiO_2-nanosheet from 250 to 300 °C. A 50 sccm flow of 1% CO, 10% O_2, and balance Ar was used for the activity measurements.

The CO oxidation activity of TiO_2-nanosheet slightly increased with the loading of 0.05 wt.% of Pt, accompanied by a slight decrease in E_a. However, the origin of this enhanced catalytic reactivity is presently unclear. It might stem from the presence of a small quantity of Pt SAs on the TiO_2 surface that did not diffuse deep into the bulk. Another possibility is the electronic interaction between Pt SAs at the subsurface and the TiO_2 surface, potentially promoting the CO oxidation activity of the TiO_2 surface.[8]

Supplementary Table 3. Comparison of E_a for CO oxidation reaction over various Pt/TiO_2 SACs.

Catalyst	E_a (kJ/mol)	Description	Reference
Pt(0.025)/TiO_2-commercial	90	Oxi. w 20% O_2 at 450 ºC, Pretreated w rtx feed at 300 ºC<br>Feed: 1% CO, 10% O_2	*Our work*
Pt(0.05)/TiO_2-truncated bipyramid	78	Oxi. w 20% O_2 at 300 ºC, Pretreated w rtx feed at 300 ºC<br>Feed: 1% CO, 10% O_2	*Our work*
TiO_2-nanosheet	138	Oxi. w 20% O_2 at 300 ºC, Pretreated w rtx feed at 300 ºC<br>Feed: 1% CO, 10% O_2	*Our work*
Pt(0.05)/TiO_2-nanosheet	113	Oxi. w 20% O_2 at 300 ºC, Pretreated w rtx feed at 300 ºC<br>Feed: 1% CO, 10% O_2	*Our work*
Pt(0.025)/TiO_2-commercial	72	Oxi. w 20% O_2 at 450 ºC, Pretreated w rtx feed at 200 ºC<br>Feed: 1% CO, 10% O_2	*1*
Pt(0.025)/TiO_2-commercial	78	Reduced w 5% H_2 at 250 ºC, Pretreated w rtx feed at 200 ºC<br>Feed: 1% CO, 10% O_2	*1*
Pt(0.025)/TiO_2-commercial	48	Reduced w 5% H_2 at 450 ºC, Pretreated w rtx feed at 200 ºC<br>Feed: 1% CO, 10% O_2	*1*
Pt(0.025)/TiO_2-commercial	69	Oxi. w 20% O_2 at 450 ºC, Pretreated w rtx feed at 200 ºC<br>Feed: 1% CO, 10% O_2	*2*
Pt(0.35)/TiO_2	56	Oxi. w 20% O_2 at 400 ºC, Pretreated w rtx feed at 300 ºC<br>Feed: 1% CO, 10% O_2	*9*
Pt(0.5)/TiO_2	39	Oxi. w 20% O_2 at 400 ºC, Pretreated w rtx feed at 400 ºC<br>Feed: 1% CO, 10% O_2	*10*

Supplementary References

1. DeRita, L. *et al.* Structural evolution of atomically dispersed Pt catalysts dictates reactivity. *Nat. Mater.* **18**, 746–751 (2019).
2. DeRita, L. *et al*. Catalyst architecture for stable single atom dispersion enables site-specific spectroscopic and reactivity measurements of CO adsorbed to Pt atoms, oxidized Pt clusters, and metallic Pt clusters on TiO_2. *J. Am. Chem. Soc.* **139**, 14150–14165 (2017).
3. https://www.thermofisher.com/us/en/home/materials-science/learning-center/periodic-table/transition-metal.html
4. Hagiwara, H., Nagatomo, M., Seto, C., Ida, S. & Ishiahara, T. Dye modification effects on TaON for photocatalytic hydrogen production from water. *Catalysts* **3**, 614–624 (2013).
5. Mom, R. *et al.* The oxidation of platinum under wet conditions observed by electrochemical X-ray photoelectron spectroscopy. *J. Am. Chem. Soc.* **141**, 6537–6544 (2019).
6. Wang, Y. *et al.* Higher loadings of Pt single atoms and clusters over reducible metal oxides: application to C–O bond activation. *Catal. Sci. Technol.* **12**, 2920-2928 (2022).
7. Wang, P. *et al.* In situ formation of cocatalytic sites boosts single-atom catalysts for nitrogen oxide reduction. *Proc. Natl. Acad. Sci. U.S.A.* **120**, e2216584120 (2023).
8. Han, B. *et al.* Strong metal-support interactions between Pt single atoms and TiO_2. *Angew. Chem. Int. Ed.* **59**, 11824 (2020).
9. Song, J. *et al.* Dispersion and support dictated properties and activities of Pt/metal oxide catalysts in heterogeneous CO oxidation. *Nano. Res.* **14**, 4841-4847 (2021).
10. Xiang, L. *et al.* Superior single-atom Pt/TiO_2 mesoporous microspheres via microdrops-confined pyrolysis/deposition for low-temperature CO oxidation. *Microporous Mesoporous Mater.* **363**, 112809 (2024).
