# Supplementary material for: Distribution of Pt single atom coordination environments on anatase TiO2 supports controls reactivity
Source: Nat Commun. 2024 Feb 2;15:998. doi: 10.1038/s41467-024-45367-z (PMC10837418; doi:10.1038/s41467-024-45367-z)
Supplement: Supplementary file 2 — Inventory of Supporting Information [file 41467_2024_45367_MOESM2_ESM.docx]

**Inventory of the Supporting Information**

| **Item** | **Description** |
| --- | --- |
| Supplementary Figure 1 | XRD patterns of TiO_2_-commercial, TiO_2_-nanosheet and TiO_2_-truncated bipyramid supports. |
| Supplementary Figure 2 | The relative ratio of (101) and (001) surfaces exposed on TiO_2_-nanosheet and TiO_2_-truncated bipyramid. |
| Supplementary Figure 3 | HAADF STEM images of Pt(0.025)/TiO_2_-commercial showing the co-existence of (101) and (001) surfaces. |
| Supplementary Figure 4 | The structure of TiO_2_-nanosheet after oxidation conditions, specifically at 300 ^o^C and 450 ^o^C. |
| Supplementary Figure 5 | HAADF STEM images showing the morphology of Pt(0.05)/TiO_2_-nanosheet. |
| Supplementary Figure 6 | HAADF STEM images showing the morphology of Pt(0.05)/TiO_2_-truncated bipyramid. |
| Supplementary Figure 7 | HAADF STEM images of Pt(0.025)/TiO_2_-commercial. |
| Supplementary Figure 8 | CO-IR spectra of Pt/TiO_2_-commercial with different Pt loadings. |
| Supplementary Discussion I | Summary of previous literature on the validation of near 100% Pt dispersion on Pt(0.025)/TiO_2_-commercial sample. |
| Supplementary Figure 9 | XPS spectra of TiO_2_-trucated bipyramid with different Pt loading, 0 wt.%, 0.05 wt.%, and 0.25 wt.%. |
| Supplementary Figure 10 | Pt 4*d* XPS spectra of Pt(0.25)/TiO_2_-nanosheet before and after Ar sputtering. |
| Supplementary Figure 11 | Pt 4*f* XPS spectra of metallic Pt and PtO_2_ with Pt valence states of 0 and +4. |
| Supplementary Figure 12 | Normalized Pt 4*d* XPS spectra of Pt(0.25)/TiO_2_-truncated bipyramid and Pt(0.25)/TiO_2_-nanosheet after sputtering with Ar_1000_ clusters. |
| Supplementary Discussion II | Estimation of the Pt oxidation state using XPS spectra. |
| Supplementary Figure 13 | HAADF STEM images of Pt(0.25)/TiO_2_-nanosheet. |
| Supplementary Figure 14 | HAADF STEM images of Pt(0.25)/TiO_2_-truncated bipyramid oxidized at 300 ^o^C. |
| Supplementary Figure 15 | IR spectra of Pt(0.25)/TiO_2_-nanosheet and Pt(0.25)/TiO_2_-truncated bipyramid collected after flowing 10% CO at 35 ^o^C for 10 min. |
| Supplementary Figure 16 | HAADF STEM images of the same region captured before and after a 1-min period of continuous irradiation. |
| Supplementary Table 1 | Atomic ratios of Ti, F and O atoms. |
| Supplementary Figure 17 | F 1*s* XPS spectra of TiO_2_-nanosheet with and without Pt. |
| Supplementary Figure 18 | XRD patterns of Pt(0.025)/TiO_2_-commercial, Pt(0.05)/TiO_2_-nanosheet, and Pt(0.05)/TiO_2_-truncated bipyramid samples before and after reduction with 10% H_2_ at 250 ^o^C for 1 hr. |
| Supplementary Figure 19 | Representative HAADF STEM images of Pt(0.25)/TiO_2_-nanosheet after reduction with 10% H_2_ at 250 ^o^C for 1 hr. |
| Supplementary Figure 20 | CO-IR spectra of Pt/TiO_2_-nanosheet and Pt/TiO_2_-truncated bipyramid with different Pt loading (0.05, 0.25 and 1 wt.%). |
| Supplementary Table 2 | Summary of the FWHM and the centroid of the IR band from CO bound to Pt SA dispersed on anatase TiO_2_ support from our work and those from literature. |
| Supplementary Figure 21 | Full-width at half maximum (FWHM) analysis using different units. |
| Supplementary Figure 22 | Dilution study for Pt(0.025)/TiO_2_-commercial. |
| Supplementary Figure 23 | The Arrhenius plots of TiO_2_-nanosheet and Pt(0.05)/TiO_2_-nanosheet. |
| Supplementary Table 3 | Comparison of $E_{a}$ for CO oxidation reaction over various Pt/TiO_2_ SACs. |
